# Senataxin RNA/DNA helicase promotes replication restart at co-transcriptional R-loops to prevent MUS81-dependent fork degradation

**DOI:** 10.1093/nar/gkae673

**Published:** 2024-08-09

**Authors:** Satyajeet Rao, Martin Andrs, Kaustubh Shukla, Esin Isik, Christiane König, Stefan Schneider, Michael Bauer, Vinicio Rosano, Jiri Prokes, Anne Müller, Pavel Janscak

**Affiliations:** Institute of Molecular Cancer Research, University of Zurich, Winterthurerstrasse 190, 8057 Zurich, Switzerland; Institute of Molecular Cancer Research, University of Zurich, Winterthurerstrasse 190, 8057 Zurich, Switzerland; Institute of Molecular Genetics of the Czech Academy of Sciences, Videnska 1083, 142 20 Prague, Czech Republic; Institute of Molecular Cancer Research, University of Zurich, Winterthurerstrasse 190, 8057 Zurich, Switzerland; Institute of Molecular Cancer Research, University of Zurich, Winterthurerstrasse 190, 8057 Zurich, Switzerland; Institute of Molecular Cancer Research, University of Zurich, Winterthurerstrasse 190, 8057 Zurich, Switzerland; Institute of Molecular Cancer Research, University of Zurich, Winterthurerstrasse 190, 8057 Zurich, Switzerland; Institute of Molecular Cancer Research, University of Zurich, Winterthurerstrasse 190, 8057 Zurich, Switzerland; Institute of Molecular Cancer Research, University of Zurich, Winterthurerstrasse 190, 8057 Zurich, Switzerland; Institute of Molecular Cancer Research, University of Zurich, Winterthurerstrasse 190, 8057 Zurich, Switzerland; Institute of Molecular Cancer Research, University of Zurich, Winterthurerstrasse 190, 8057 Zurich, Switzerland; Institute of Molecular Genetics of the Czech Academy of Sciences, Videnska 1083, 142 20 Prague, Czech Republic

## Abstract

Replication forks stalled at co-transcriptional R-loops can be restarted by a mechanism involving fork cleavage-religation cycles mediated by MUS81 endonuclease and DNA ligase IV (LIG4), which presumably relieve the topological barrier generated by the transcription-replication conflict (TRC) and facilitate ELL-dependent reactivation of transcription. Here, we report that the restart of R-loop-stalled replication forks *via* the MUS81-LIG4-ELL pathway requires senataxin (SETX), a helicase that can unwind RNA:DNA hybrids. We found that SETX promotes replication fork progression by preventing R-loop accumulation during S-phase. Interestingly, loss of SETX helicase activity leads to nascent DNA degradation upon induction of R-loop-mediated fork stalling by hydroxyurea. This fork degradation phenotype is independent of replication fork reversal and results from DNA2-mediated resection of MUS81-cleaved replication forks that accumulate due to defective replication restart. Finally, we demonstrate that SETX acts in a common pathway with the DEAD-box helicase DDX17 to suppress R-loop-mediated replication stress in human cells. A possible cooperation between these RNA/DNA helicases in R-loop unwinding at TRC sites is discussed.

## Graphical Abstract

**Graphical Abstract:**
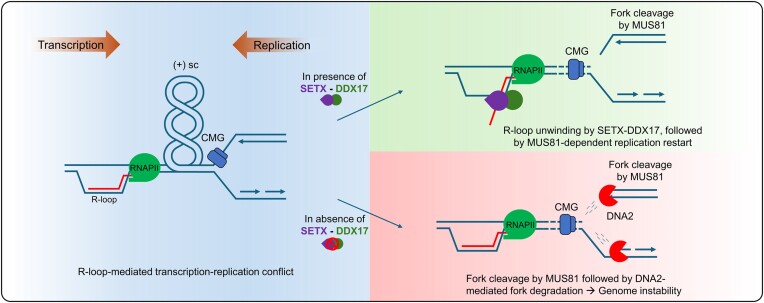

## Introduction

Complete replication of the genetic material is of utmost importance to achieve proper chromosome segregation. However, DNA replication is constantly challenged by various endogenous and exogenous factors that can impede replication fork progression, leading to DNA replication stress (1). Cells have evolved specialized mechanisms to resolve any errors in the DNA replication process, and disruption of these processes can lead to genomic instability and carcinogenesis (1,2).

Replication fork obstruction can be caused by transcription complexes trapped on the DNA due to the formation of an R-loop (3). R-loops are three-stranded structures formed co-transcriptionally by annealing of the nascent transcript to the template DNA strand in the underwound region behind the transcription complex, which leaves the non-template strand exposed as a single-stranded DNA (ssDNA) loop (4). R-loops tend to form in regions where the non-template strand can fold into G-quadruplex (G4) structures, which presumably stabilize the R-loop and promote its extension (4–6). Formation of R-loops in these G-rich regions is also promoted by head-on collisions of active transcription complexes with the replisome, particularly, if the progression of the latter is slowed down (7–9).

Replication fork stalling induced by R-loop forming transcription complexes is an active process involving RAD51-mediated fork reversal (10). In this fork remodeling reaction, which also requires the DNA translocases ZRANB3, HLTF and SMARCAL1, the nascent DNA strands unwound by the re-annealing of the parental strands form the so-called regressed arm that is covered by BRCA2-stabilized RAD51 filament to prevent nascent DNA resection by MRE11 nuclease (2). However, these R-loop-stalled forks can be reactivated in a multi-step process triggered by the action of two DNA helicases, RECQ1 and RECQ5 (10). The RECQ1 helicase mediates reverse branch migration to eliminate reversed forks, while the RECQ5 helicase removes RAD51 from the stalled fork to prevent fork reversal and promote fork cleavage by MUS81 endonuclease (10–12). This initiates the replication restart process that additionally requires the strand-annealing factor RAD52, the DNA ligase IV (LIG4)/XRCC4 complex, the RNA polymerase II (RNAPII) elongation factor ELL, the DNA-directed primase/polymerase PRIMPOL, and the non-catalytic subunit of DNA polymerase delta POLD3 (9,10). It is proposed that MUS81-mediated cleavage of the leading arm of the stalled fork relieves the topological barrier generated by the TRC, thereby enabling ELL-dependent reactivation of transcription (10). Subsequent fork re-ligation by RAD52 and the LIG4/XRCC4 complex allows the passage of the reactivated transcription complex to the lagging arm of the fork, after which DNA replication is restarted by PRIMPOL and POLD3 (9,10).

This sequential restart of RNA and DNA synthesis at sites of R-loop-mediated TRCs also requires the removal of the R-loop and G4 structures, which would otherwise impede the progression of the reactivated replication fork. Our recent studies have identified the mismatch repair protein MutSβ, an MLH1–PMS1 heterodimer termed MutLβ, and the G4-resolving helicase FANCJ as factors that act in a coordinated fashion to promote the restart of R-loop-stalled fork *via* the MUS81–LIG4–ELL axis (13). In addition, we have found that this DNA-repair process requires the DEAD-box helicase DDX17 that can efficiently unwind RNA:DNA hybrids in short R-loop structures (14). Our findings suggest a model wherein MutSβ and MutLβ mediate FANCJ recruitment to G4s within R-loops at TRC sites for G4 unwinding. This facilitates the loading of DDX17 on the ssDNA loop which is required to initiate unwinding of the RNA:DNA hybrid in the R-loop (13). However, the DEAD-box helicases can only unwind short duplexes (15), whereas RNA:DNA hybrids in R-loops can be as long as ∼1000 bp in human cells (16), suggesting that a processive RNA/DNA helicase may be required for the unwinding of R-loops at TRC sites to allow replication restart *via* the MUS81–LIG4–ELL axis.

Senataxin (SETX) is a superfamily 1B helicase, which is capable of unwinding RNA:DNA hybrids with a 5′ to 3′ polarity (17), and fulfils multiple roles in the metabolism of R-loops in human cells (18,19). Mutations in the *SETX* gene are associated with the progressive neurological disorders Amyotrophic lateral sclerosis 4 (ALS4) and Ataxia with oculomotor apraxia 2 (AOA2) (20,21), highlighting the importance of SETX functions in the cell. SETX is known to unwind R-loops formed at the transcription pause sites downstream of gene poly(A) signals to promote Xrn2-mediated termination of RNAPII transcription (22). SETX also removes R-loops near the promoters of RNAPII-transcribed genes to prevent transcription stress and chromosomal rearrangements (23). The recruitment of SETX to R-loops at transcription pause sites is mediated through its physical interaction with BRCA1, and disruption of this complex leads to R-loop-dependent DNA damage at these loci (24). Interestingly, the yeast SETX homolog, Sen1, has also been shown to associate with replisomes to promote replication fork progression across RNAPII-transcribed genes, thereby maintaining chromosome stability (25,26). Although evidence suggests that SETX accumulates at sites of R-loop-mediated TRCs and prevents transcription-dependent replication stress in human cells (27–29), its role in TRC resolution remains unclear. Here, we reveal that SETX counteracts R-loop-mediated TRCs by promoting replication restart *via* the MUS81–LIG4–ELL pathway in a manner dependent on its helicase activity. Furthermore, we show that SETX deficiency leads to MUS81-dependent degradation of nascent DNA strands at R-loop-stalled forks and R-loop-dependent chromosomal instability due to persistence of underreplicated DNA. Our data suggest that SETX functions in conjunction with DDX17 to remove R-loops at TRC sites, thereby facilitating replication restart.

## Materials and methods

### Plasmid constructions

SETX open reading frame (ORF) was amplified by PCR from the plasmid pCMVFLAG3-SETX (MRC PPU Reagents, DU 42219) and cloned into pAIO vector between BspEI and Acc65I sites to generate a SETX-3xFLAG fusion under control of CMV enhancer/promoter followed by two tet operators (30). Site-directed mutagenesis was used to introduce silent mutations in SETX ORF to confer resistance to SETX siRNA #1 (siSETX-1). The resulting construct (pAIO-SETX-FLAG) was used as a template for another round of site-directed mutagenesis to introduce K1969R substitution in helicase motif I of SETX [pAIO-SETX(K1969R)-FLAG]. The sequences of DNA primers used for plasmid constructions are listed in Supplementary Table S1.

### Cell culture

U2OS (HTB-96), U2OS *SETX* knockout and HEK293 (CRL-1573) cells were grown in Dulbecco's modified Eagle's medium (DMEM; Gibco, 41966-029), supplemented with 10% fetal bovine serum (FBS; Gibco, 10270-106) and penicillin/streptomycin (100 units/ml; Sigma Aldrich, P4333-100ML), at 37°C in a water-jacketed incubator supplied with 5% CO_2_. U2OS T-REx cell lines expressing RNaseH1-GFP, RNaseH1(D210N)-GFP, SETX-FLAG and SETX(K1969R)-FLAG, respectively, were grown in DMEM supplemented with 10% tetracycline-free FBS, penicillin/streptomycin (100 units/ml), 1μg/ml puromycin (InvivoGen, ant-pr-1) and 50 μg/ml hygromycin B (InvivoGen, ant-hg-1). Expression of SETX-FLAG was induced by addition of 10 ng/ml doxycycline (Takara Bio, 631 311) for 24 h. Expression of RNaseH1-GFP was induced by addition of 1 ng/ml of doxycycline for 24 h. Human hTERT-immortalized human fibroblasts 1BR (control) and 411BR (isolated from a LIG4 syndrome patient) were grown in DMEM supplemented in 15% FBS and penicillin/streptomycin (100 units/ml) (31).

### Generation of stable cell lines

U2OS T-REx cells grown in 6-cm plates were transfected with 2 μg of pAIO-SETX-FLAG or pAIO-SETX(K1969R)-FLAG vector using TransIT-X2 transfection reagent (Mirus, MIR 6003). Puromycin was added to transfected cells to a final concentration of 1 μg/ml 48 h post transfection, and to non-transfected cells as control. Transfected cells were selected in puromycin until non-transfected cells died (48–72 h). The surviving cellular pool was seeded to achieve single-cell separation and was allowed for colony formation for 10–14 days. Isolated clones were collected under a light microscope. Individual clones were allowed to proliferate and screened for appropriate expression by western blot and immunofluorescence with anti-FLAG and anti-Senataxin antibodies.

### Generation of *SETX* knockout cells

U2OS *SETX* knockout cell lines were generated using CRISPR/Cas9 technology as follows: sgRNA target sequence (CGTTCATGTAGAAGCAAGTA) was cloned into pX330-U6-Chimeric_BB-CBh-hSpCas9 vector (Addgene plasmid # 42 230) using the BbsI site, and verified by sequencing. The resulting construct was transfected to U2OS cells using TransIT-X2 transfection reagent. Neomycin (500 μg/ml; InvivoGen, ant-gn-1) was added to transfected and non-transfected cells 48 h post-transfection. After 48–72 h, surviving pools were split in two halves. One portion was used to analyze the presence of indel mutations via T7 endonuclease I assay (New England Biolabs, M0302S), and the remaining cells were seeded to achieve single-cell separation. After 10–14 days, individual clones were isolated using a light microscope. Isolated clones were allowed to proliferate and screened for SETX expression by western blot using anti-Senataxin antibody. The *SETX* knockout cell line was transfected with pAIO-SETX-FLAG vector for complementation, or with pAIO empty vector as control, using TransIT-X2 transfection reagent. Transfected cells were selected with 1 μg/ml puromycin until non-transfected cells had died (48–72 h). Surviving cells transfected with pAIO plasmid were cultured as a pool with 1 μg/ml puromycin. Surviving cells transfected with pAIO-SETX-FLAG were diluted and reseeded. After 6–8 days cells had grown into small colonies which were isolated and allowed to proliferate. Clones were screened for the expression of SETX-FLAG by western blot and immunofluorescence using anti-FLAG antibody.

### siRNA transfections

Exponentially growing cells were transfected with 40 nM siRNA using Lipofectamine RNAiMAX (Invitrogen, 13 778 150) according to the manufacturer's protocol. Fresh medium was added 24 h after transfection and cells were used for experiment 72 h after transfection. For complementation studies, SETX-FLAG and SETX(K1969R)-FLAG expressing cells were seeded 24 h post siSETX-1 transfection, and 10 ng/ml doxycycline was added 48 h post-transfection. Experiments were performed 72 h post-transfection. The sequences of the sense strand of siRNA duplexes used in this study are shown in Supplementary Table S2.

### Western blot analysis

Exponentially growing cells were harvested by trypsinization, washed with 1x PBS (phosphate buffer saline; 137 mM NaCl, 2.7 mM KCl, 10 mM Na_2_HPO_4_ and 1.8 mM KH_2_PO_4_), resuspended in Lysis Buffer [40 mM Tris–HCl (pH 7.5), 5 mM MgCl_2_, 2% (w/v) SDS] supplemented with 1 mM PMSF (phenylmethanesulphonylfluoride; Sigma-Aldrich, P7626), protease inhibitor cocktail (cOmplete EDTA-free; Roche, 4 693 132 001) and phosphatase inhibitor cocktail (PhosSTOP, Roche, 4 906 837 001), and incubated at room temperature (RT) for 10 min. Cells were then sonicated for 5 min at high intensity (30 s ON, 30 s OFF) in a bath sonicator. Samples were denatured by boiling at 95°C for 10 min in the presence of Laemmli buffer [0.5 M Tris–HCl (pH 6.8), 0.01% (w/v) Bromophenol blue, 0.1 M DTT, 10% (v/v) glycerol, 2% (w/v) SDS] and loaded onto 6% or 10% SDS-polyacrylamide gels (SDS-PAGE). After electrophoresis, resolved proteins were transferred to a Hybond-P PVDF membrane in a wet transfer apparatus (BioRad Mini-Trans-Blot Cell, 1 703 930) containing transfer buffer [25 mM Tris, 190 mM Glycine and 0.01% (w/v) SDS] at 30 V, overnight in cold room. Following transfer, membranes were stained with Ponceau-S stain for 5 min to confirm the efficient transfer. Membranes were then de-stained by washing with TBS-T [20 mM Tris–HCl (pH 7.4), 150 mM NaCl and 0.2% (v/v) Tween-20] and blocked in 5% (w/v) dry milk in TBS-T for 1 hour. Following blocking, membranes were incubated with primary antibodies diluted in 5% (w/v) dry milk/TBS-T overnight at 4°C. After three washes with TBS-T, membranes were incubated with horseradish peroxide (HRP)-conjugated secondary antibodies for 1 hour at 4°C. Membranes were then washed thrice with TBS-T and bands were developed by detection of chemiluminescence signal after adding the HRP substrates (hydrogen peroxide and luminol; Thermo Scientific SuperSignal West Femto Maximum Sensitivity Substrate, 34 096). Antibodies used: Senataxin (rabbit polyclonal, Bethyl, A301-104A; 1:1000), Phospho-CHK1 (Ser345) (rabbit polyclonal, Cell Signalling, 2341S, 1:1000), CHK1 (mouse monoclonal, Santa Cruz Biotechnology, sc-515369, 1:500), FLAG (mouse monoclonal, Sigma, F1804; 1:1000), TFIIH (rabbit polyclonal, Santa Cruz Biotechnology, sc-293; 1:2000), GAPDH (mouse monoclonal, Santa Cruz Biotechnology, sc-47724; 1:2000), MUS81 (mouse monoclonal, Santa Cruz Biotechnology, sc-53382; 1:500), GFP (rabbit polyclonal, Abcam, ab290; 1:2000), BRCA2 (mouse monoclonal, Sigma, OP95; 1:500), ZRANB3 (rabbit polyclonal, LindCare, 23111–1-AP; 1:1000), DDX17 (mouse monoclonal, Santa Cruz Biotechnology, sc-398168; 1:500), goat anti-rabbit IgG HRP (Sigma A0545; 1:10 000) and goat anti-mouse IgG HRP (Sigma A4416; 1:10 000).

### Detection of R-loops by monitoring RNaseH1(D210N)-GFP foci

U2OS T-REx cells expressing RNaseH1(D210N)-GFP were grown on sterile glass coverslips. Expression of RNaseH1(D210N)-GFP was induced by addition of 1 ng/ml doxycycline for 24 h. Coverslips were washed twice with ice-cold 1X PBS and pre-extraction was done on ice with ice-cold buffer [25 mM HEPES (pH 7.4), 50 mM NaCl, 1mM EDTA, 3 mM MgCl_2_, 300 mM sucrose and 0.5% (v/v) Triton X-100] for 10 min. Coverslips were washed twice with PBS and cells were fixed with 4% (v/v) formaldehyde/PBS (Sigma Aldrich, F8775-500ML) for 15 min in dark at RT. Cells were washed thrice with PBS and an additional fixation step was carried out with freezing-cold methanol (100%) at −20°C for 15 min. Coverslips were washed three times with PBS, and were blocked with 3% (w/v) BSA in PBS for 30 min at RT. After blocking, coverslips were incubated with anti-PCNA antibody dissolved in blocking solution for 2 h at RT, in a humidified chamber. After three PBS washes, coverslips were incubated with secondary antibody for 1 hour at RT in dark. Cells were then washed three times with PBS before counterstaining with DAPI (0.5 μg/ml) for 10 min at RT in dark. Coverslips were washed twice with PBS and mounted on glass slides using Fluoromount-G (Invitrogen, 00-4958-02). Images were captured at 63X magnification on a Leica microscope model DM6B, coupled to a DMC 2900 digital camera. In each experiment, at least 200 PCNA^+^ and 200 PCNA^−^ nuclei were analyzed for the presence of RNaseH1(D210N)-GFP foci using ImageJ find maxima tool. Antibodies used: PCNA (rabbit polyclonal, Abcam, ab18197; 1:1000), secondary goat anti-rabbit Alexa 594 (Thermo Fisher Scientific, A11037; 1:500).

### DNA fiber spreading assay

Exponentially growing cells were pulse-labeled with 5-chloro-2′-deoxyuridine (CldU, 25 μM; Sigma-Aldrich, C6891) for 30 min at 37°C. Following three quick washes with pre-warmed PBS, cells were pulse-labeled with 5-iodo-2′-deoxyuridine (IdU, 250 μM; Sigma-Aldrich, I7125) at 37°C for 30 min. Cells were washed with PBS and harvested by trypsinization. Centrifugation was done to pellet the cells and the pellet was washed with ice-cold PBS. Cells were then resuspended in ice-cold PBS in a concentration of 2.5 × 10^5^ cells/ml. Glass slides were wiped once with kimwipes and left to air dry for 2 min. 7.5 μl of lysis buffer [200 mM Tris–HCl (pH 7.5), 50 mM EDTA and 0.5% (w/v) SDS] were spotted on the top-center of the glass slide and 2.5 μl of the cell suspension was added to the lysis buffer drop. The solution was mixed by up and down pipetting three times and left undisturbed for 9 min at RT. Slides were then tilted at 15–20° to allow lysed cells to slowly move down resulting in the spreading of DNA fibers along the length of the glass slide. The slides were air dried at RT and fixed in methanol/acetic acid solution (3:1) at RT for 15 min. After washing thrice with PBS, DNA fibers were denatured by keeping the glass slides submerged in 2.5 N HCl for 1 h at RT. Following three PBS washes, slides were kept in blocking solution [2% (w/v) BSA/0.1% (v/v) Tween-20/PBS] 30 min at RT. Subsequently, DNA fibers were incubated with primary antibodies diluted in blocking solution overnight at 4°C in a humidified chamber. Slides were washed thrice with PBS-T (PBS supplemented with 0.2% (v/v) Tween-20] for 5 min each wash, and incubated with secondary antibodies for 1 hour at RT. Following this, slides were washed again with PBS-T thrice for 5 min each, air dried and mounted using ProLong Gold Antifade mounting medium (Invitrogen, P36930). DNA fiber images were captured at 63X magnification on a Leica microscope model DM6B, coupled to a DMC 2900 digital camera. Lengths of replication tracts on DNA fibers were measured manually using the segmented line tool in ImageJ. Antibodies used: CldU (rat monoclonal anti-BrdU, abcam, ab6326; 1:500), IdU (mouse monoclonal anti-BrdU, BD Biosciences, 347 580; 1:100), secondary donkey anti-rat Cy3 (Jackson ImmunoResearch, 712-166-153; 1:150) and secondary goat anti-mouse Alexa 488 (Thermo Fisher Scientific, A11001; 1:300).

### *In situ* proximity ligation assay

Cells growing on coverslips were pulse-labeled with 5-ethynyl-2′-deoxyuridine (EdU, 10 μM; Thermo Fisher Scientific, A10044) for 15 min to mark replicating cells. Cells were briefly washed twice with PBS and pre-extracted with ice-cold pre-extraction buffer [0.5% (v/v) Triton X-100/PBS containing protease inhibitor cocktail] for 10 min on ice. After three PBS washes, cells were fixed with 4% (v/v) formaldehyde/PBS for 15 min at RT in dark. Cells were washed thrice with PBS and a subsequent fixation step was carried out with freezing-cold methanol (100%) at −20°C for 15 min. Following three PBS washes, blocking was done with 3% (w/v) BSA/PBS for 30 min at RT. Click reaction was performed for 30 min at RT according to the manufacturer's protocol (Click-iT Plus Alexa Fluor 488 Imaging kit; Invitrogen C10337) to detect the EdU-marked S-phase cells. Cells were then washed thrice with PBS and incubated with primary antibodies diluted in blocking solution at 4°C overnight. The next morning, cells were washed thrice with PBS, and PLA was performed according to the manufacturer's protocol (Duolink^®^ Proximity Ligation Assays). Coverslips were incubated with anti-mouse MINUS (Sigma Aldrich, DUO92004-100RXN) and anti-rabbit PLUS (Sigma Aldrich, DUO92002-100RXN) PLA probes for 1 hour at 37°C. Coverslips were then washed thrice with wash buffer A [0.01M Tris–HCl (pH 7.4), 0.15 M NaCl and 0.05% (v/v) Tween-20] for 5 min each with gentle agitation. A ligation step was performed for 30 min at 37°C. Following ligation, coverslips were washed twice with wash buffer A for 2 min each with gentle agitation. The amplification step was carried out with Duolink In situ Detection Reagents Red (Sigma Aldrich, DUO92008-100RXN) for 100 min at 37°C. Coverslips were then washed twice with wash buffer B [0.2 M Tris–HCl (pH 7.5), 0.1 M NaCl] for 10 min each wash, with gentle agitation. This was followed by one wash with 0.01× buffer B and co-staining with DAPI (0.5 μg/ml) and mounting on glass slides with Fluoromount-G mounting media. Images were captured at 63× magnification on a Leica microscope model DM6B, coupled to a DMC 2900 digital camera. In each experiment, at least 200 nuclei were analyzed for the presence of PLA foci using ImageJ find maxima tool. Antibodies used: PCNA (rabbit polyclonal, Abcam, ab18197; 1:1000), RNA Polymerase II (H5) (mouse monoclonal, Biolegend, 920 204; 1:1000), Senataxin (rabbit polyclonal, Bethyl, A301-104A; 1:500) and DDX17 (mouse monoclonal, Santa Cruz Biotechnology, sc-398168; 1:1000).

### Detection of RNA:DNA hybrids by DNA-RNA immunoprecipitation

Cells were grown in a single well of a 6-well plate following depletions. Cells were harvested by trypsinization followed by centrifugation. The cell pellet was washed with ice-cold PBS and resuspended in lysis buffer [10 mM Tris–HCl (pH 8), 100 mM NaCl, 25 mM EDTA, 0.5% (v/v) SDS and 10 μg/ml proteinase K] and incubated at 37°C overnight on a rotary shaker. Phenol-chloroform extraction of nucleic acids was performed, and genomic DNA was precipitated using isopropanol, followed by washing the genomic DNA pellet with 80% (v/v) ethanol. The DNA pellet was allowed to air dry and dissolved in 500 μl of autoclaved H_2_O. Genomic DNA fragmentation was performed with a mixture of restriction enzymes (EcoRI, BamHI, BsrGI, HindIII and XhoI) at 37°C for 3 h on a rotary shaker. DNA digestion was confirmed by agarose gel electrophoresis. The sample was divided in two halves, which were incubated with or without 40 units of RNaseH enzyme (New England Biolabs, M0297L) overnight at 37°C with rotation. Phenol-chloroform extraction was performed, and DNA was precipitated as described above. 10 μg of the purified DNA was dissolved in 500 μl binding buffer [10 mM Na_3_PO_4_ (pH 7), 0.15 M NaCl and 0.05% (v/v) Triton X-100]. 1/10 volume of this solution (50 μl) was taken in a separate tube to serve as input (10%). The samples were incubated with 6 μg of S9.6 antibody overnight at 4°C with rotation. The samples were then incubated with 35 μl of protein A/G magnetic beads (Thermo Scientific Pierce Protein A/G Magnetic beads, 88 802) at 4°C for 2 h with rotation. Beads were collected on a magnetic rack and washed twice with binding buffer (15 min/wash with rotation), followed by one wash with TE buffer [10 mM Tris (pH 8), 1 mM EDTA]. Washed beads were incubated with 500 μl of elution buffer [50 mM Tris (pH 8), 10 mM EDTA, 0.5% SDS and 10 μg/ml proteinase K] at 55°C for 45 min. Beads were separated on a magnetic rack and supernatant was collected. Phenol-chloroform extraction and DNA precipitation were performed as described above. The DNA pellet was allowed to air dry and then dissolved in 50 μl of autoclaved H_2_O. Quantitative real-time PCR (qPCR) was performed with 2 μl of each sample using the SYBR Green master mix (Roche LightCycler 480 SYBR Green I Master, 04 707 516 001) on a Roche LightCycler 480 instrument II (Roche, 05 015 278 001). The following targets were analyzed for RNA:DNA hybrid enrichment: APOE, RPL13A and BTBD19. SNRPN was used as a negative control. Following qPCR, the percentage of input was calculated using the formula: % of input = 100 × 2[Ct input(corrected) – Ct DRIPed DNA], where Ct (cycle threshold) input (corrected) = Ct input – log_2_(10). log_2_(10) represents 1/10th of the immunoprecipitated DNA (10% of the sample was taken as input). The sequences of DNA primers used for qPCR are listed in Supplementary Table S3.

### Cytokinesis-block micronucleus assay

Cells growing on coverslips were treated with Cytochalasin B (2 μg/ml; Sigma Aldrich, C6762) for 16 h to block cytokinesis. Coverslips were washed twice with PBS and fixed in 4% (v/v) formaldehyde/PBS for 15 min in dark. Nuclei were stained with DAPI (0.5 μg/ml) for 10 min at RT and coverslips were mounted on glass slides using Fluoromount-G mounting medium. Images were acquired automatically on an IX83 microscope (Olympus) equipped with ScanR imaging platform using 40X/0.9 NA objective. At least 300 binucleated cells were scored for the presence of micronuclei using ImageJ multi-point tool.

### Analysis of mitotic DNA synthesis

Cells growing in 10-cm dishes were treated with CDK1 inhibitor, RO-3306 (9 μM; Sigma, SML0569-5MG) for 16 h to block mitotic entry. Cells were then washed quickly with pre-warmed medium followed by 1 h incubation with fresh medium containing 20 μM EdU and 0.1 μg/ml Colcemid (Gibco, 15 210 040), which blocks cells in metaphase. The medium was collected in a 15 ml tube, and the plate was washed gently once with PBS, which was also collected in the same tube. Finally, the attached cells were harvested into the same tube by trypsinization. Cells were centrifuged and resuspended in fresh PBS, and then centrifuged again. 5 ml of pre-warmed KCl solution (75 mM) was added dropwise to the cell pellet, while gently vortexing, followed by incubation at 37°C for 20 min. 1.25 ml of fixation solution (methanol/acetic acid, 3:1) was added, mixed gently and incubated again at 37°C for 10 min. Swollen cells were centrifuged, and the pellet was resuspended in 5 ml of fixation solution added in a dropwise fashion, while gently vortexing, followed by centrifugation. This step was repeated a total of three times. Fixed and swollen cells were then resuspended in 500 μl of fixation solution and dropped on pre-cooled glass slides. Slides were allowed to air dry and blocked in 3% (w/v) BSA/PBS for 30 min at RT. Click reaction was performed with the Click-iT Plus Alexa Fluor 488 Imaging kit as described above. After the click reaction, slides were washed briefly with blocking solution, followed by two washes with PBS. Slides were then counter-stained with DAPI (0.5 μg/ml) for 10 min at RT. Slides were washed thrice with PBS and mounted using Prolong Gold mounting medium. Images were captured at different Z-planes at 63× (oil objective) magnification on Leica microscope model DM6B, coupled to the DMC 2900 digital camera. At least 50 metaphase spreads were scored for the presence of EdU foci using ImageJ.

### Analysis of 53BP1 nuclear bodies

Cells growing on coverslips were pulse-labeled with EdU (10 μM) for 15 min and then washed twice with PBS and fixed with 4% (v/v) formaldehyde/PBS. Click reaction was performed for 30 min at RT according to the manufacturer's protocol (Click-iT Plus Alexa Fluor 488 Imaging kit; Invitrogen, C10337) to detect the EdU-marked S-phase cells. Following blocking in 3% (w/v) BSA/PBS for 30 min at RT, coverslips were incubated with anti-53BP1 antibody (diluted in blocking solution) for 2 h at RT, in a humidified chamber. After three PBS washes, coverslips were incubated with secondary antibody for 1 h at RT in dark. Coverslips were then washed thrice with PBS and counter-stained with 0.5 μg/ml DAPI, followed by mounting on glass slides using Fluoromount-G mounting medium. Images were captured automatically on an IX83 microscope (Olympus) equipped with ScanR imaging platform using 40X/0.9 NA objective. At least 300 EdU-negative cells were scored for the presence of 53BP1 nuclear bodies using ImageJ. Antibodies used: rabbit polyclonal 53BP1 (Abcam, ab36823; 1:200), secondary goat anti-rabbit Alexa 594 (Thermo Fisher Scientific, A11037; 1:500).

### Co-immunoprecipitation assay

Untreated or CPT-treated HEK293 cells were harvested and washed with cold PBS twice. The cell pellet was suspended in cold IP lysis buffer [50 mM Tris–HCl (pH 7.4), 150 mM NaCl, 1 mM EDTA, 1% (v/v) NP-40, 0.2% (v/v) Triton X-100, 5% (v/v) glycerol and 1mM DTT], supplemented with Roche cOmplete Protease Inhibitor Cocktail, Roche PhosSTOP, and 20 mM *N*-ethylmaleimide (Sigma-Aldrich, E3876). The cell suspension was incubated on a rotator for 30 min at 4°C and then subjected to sonication on ice (Q-sonica, model Q125; 10 cycles of 10 s ON and 30 s OFF at 35% amplitude). Samples were centrifuged at 16 000g for 30 min at 4°C. Protein concentration of the collected supernatant was estimated using Bicinchoninic acid assay (Sigma-Aldrich, BCA1-1KT) and a fraction of the supernatant was saved as input. Subsequently, 1200 μg of protein from the supernatant was incubated with 5 μg of rabbit polyclonal anti-SETX antibody (Bethyl, A301-104A), 250 units of benzonase nuclease (Sigma-Aldrich, E1014) and 5 mM MgCl_2_ on a rotator at 4°C. After 4 h of incubation, 25 μl of Pierce™ ChIP-grade Protein A/G Magnetic Beads (Cat. No. 26 162), washed and equilibrated with IP lysis buffer, were added to each sample and incubated at 4°C on a rotator for 2 h. Finally, the beads were magnetically separated and washed 3 times with IP lysis buffer and resuspended in SDS-PAGE sample loading buffer and subjected to western blot analysis.

### Statistical analysis

Statistical analysis was performed with GraphPad Prism 10 software using either two-tailed non-parametric Kruskal–Wallis test followed by Dunn's multiple comparisons test or ordinary one-way analysis of variance (ANOVA) followed by Šídák's multiple comparisons, with a single pooled variance, where appropriate. Details of statistics in each experiment can be found in the corresponding figure legends.

## Results

### SETX-depleted cells exhibit R-loop-mediated replication stress

**Figure 1.**
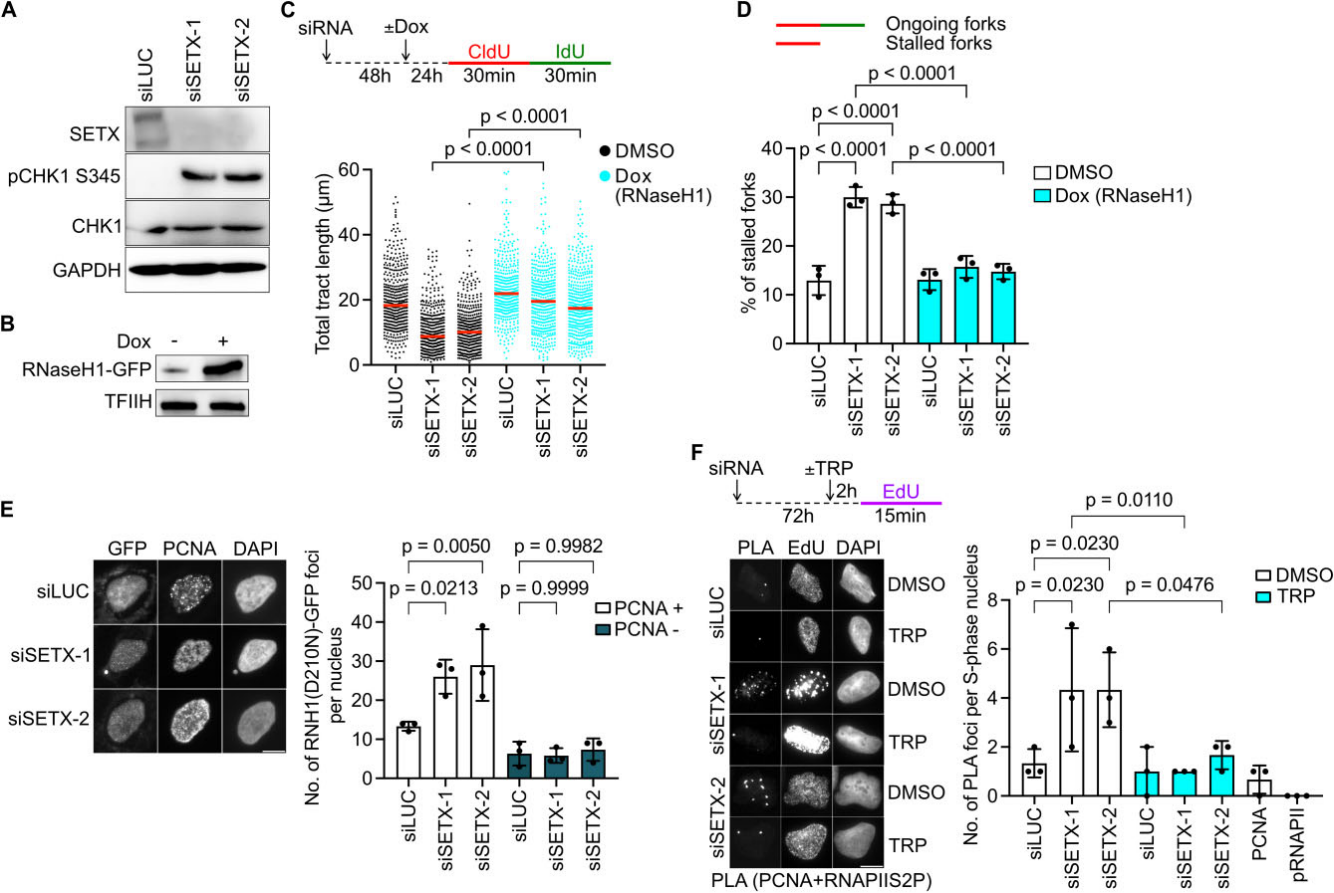
SETX-depleted cells exhibit R-loop-mediated replication stress. (**A**) Western blot analysis to check SETX expression levels and CHK1-S345 phosphorylation in U2OS cells transfected with two different siRNAs against SETX for 72 h. (**B**) Western blot analysis to confirm over-expression of RNaseH1-GFP in U2OS T-REx [RNaseH1-GFP] cells treated with doxycycline (Dox; 1 ng/ml) for 24 h. (**C**) Schematic of DNA fiber labeling and quantification of replication tract lengths in mock (siLUC)- and SETX-depleted U2OS T-REx [RNaseH1-GFP] cells. 1 ng/ml Dox was added 24 h before the labeling to induce RNase H1-GFP over-expression. Dimethylsulphoxide (DMSO) was used as a vehicle. The values of CldU + IdU tract lengths measured in three independent experiments are plotted (n > 300). Red lines represent the median. (**D**) Quantification of replication fork stalling events on DNA fibers in (**C**). Replication tracts containing only the first label (CldU-only tracts) were designated as stalled replication forks while the replication tracts containing both labels (CldU + IdU) were scored as ongoing forks. Data represent mean ± SD (*n* = 3). (**E**) Representative images and quantification of RNaseH1(D210N)-GFP foci in U2OS T-REx [RNaseH1(D210N)-GFP] cells transfected with indicated siRNAs. PCNA immunofluorescence staining was used to identify S-phase cells (PCNA^+^). (**F**) Representative images and quantification of PLA foci between PCNA and elongating form of RNA polymerase II (RNAPIIS2P) in U2OS cells transfected with indicated siRNAs. EdU incorporation was performed to mark S-phase cells. Triptolide (1 $\mu$M) was added 2 h prior to PLA. (E, F) Medians of data sets from three independent experiments are plotted (n > 200). Data are presented as mean ± SD. Scale bar, 10 $\mu {\mathrm{m}}$. Statistical analysis: Kruskal–Wallis test followed by Dunn's multiple comparisons test was used in (**C**). Ordinary one-way ANOVA followed by Šídák's multiple comparisons, with a single pooled variance was used in (D)–(F).

The yeast SETX homolog, Sen1, has been reported to aid the replication of highly transcribed regions by avoiding R-loop accumulation (25), and *sen1* cells were found to accumulate DNA damage (32). To explore the role of human SETX in promoting DNA replication, we knocked down its expression in U2OS cells using two different small-interfering RNAs (siRNAs) and analyzed the markers of DNA replication stress. We observed that SETX depletion increased the phosphorylation of CHK1 at S345 (Figure 1A), which is a bona fide target of ATR and an indicator of replication stress (33). To get a direct readout of replication stress, we assessed the replication dynamics by performing DNA fiber spreading assay based on pulse labeling replication tracts with halogenated thymidine analogs, CldU and IdU, followed by their visualization on DNA fiber spreads by indirect immunofluorescence (34). We observed a significant decrease in replication fork speed in SETX-depleted cells as compared to cells transfected with control siRNA (Supplementary Figure S1A). To determine whether the impaired replication fork progression caused by SETX deficiency is a consequence of R-loop accumulation, we depleted SETX in a U2OS T-REx cell line conditionally over-expressing RNaseH1, an enzyme that cleaves the RNA moiety in RNA:DNA hybrids thereby removing R-loops (10). DNA fiber experiments with these cells revealed that the replication fork slowdown induced by SETX depletion was rescued after over-expression of RNaseH1, suggesting that it is caused by R-loops (Figure 1B, C). We then examined the frequency of replication fork stalling events by quantifying the percentage of CldU tracts that were not followed by an IdU tract. The tracts containing both labels were considered as ongoing forks. We found that SETX-depleted cells exhibited increased accumulation of stalled replication forks, which was suppressed by over-expression of RNaseH1, suggesting R-loop dependence (Figure 1D; Supplementary Figure S1B).

To corroborate that SETX depletion induces R-loop accumulation, we made use of a U2OS T-REx cell line inducibly expressing a GFP-tagged RNaseH1 variant, which is made catalytically inactive by a point mutation (D210N) in its nuclease domain (35). As expected, we observed an increased accumulation of RNaseH1(D210N)-GFP foci in SETX-depleted cells as compared to mock-depleted cells (Figure 1E). Interestingly, this was seen in PCNA-positive cells but not in PCNA-negative cells (Figure 1E), suggesting that SETX suppresses R-loop accumulation specifically during S-phase. R-loops can form as a consequence of head-on TRCs, leading to replication fork stalling (8,9). To visualize persistent TRCs *in vivo*, we monitored colocalization between PCNA (a part of the replisome) and the elongating form of RNAPII (RNAPIIS2P, where S2P stands for phosphorylation of serine-2 in the C-terminal repeat domain of RNAPII) by proximity ligation assay (PLA) (8,10). We observed that SETX-depleted U2OS cells exhibited a marked increase in the frequency of PCNA/RNAPIIS2P PLA foci as compared to mock-depleted cells (Figure 1F), suggesting a defect in TRC resolution. Of note, the formation of these PLA foci was abolished by inhibition of transcription with triptolide, confirming that they represent TRCs (Figure 1F).

**Figure 2.**
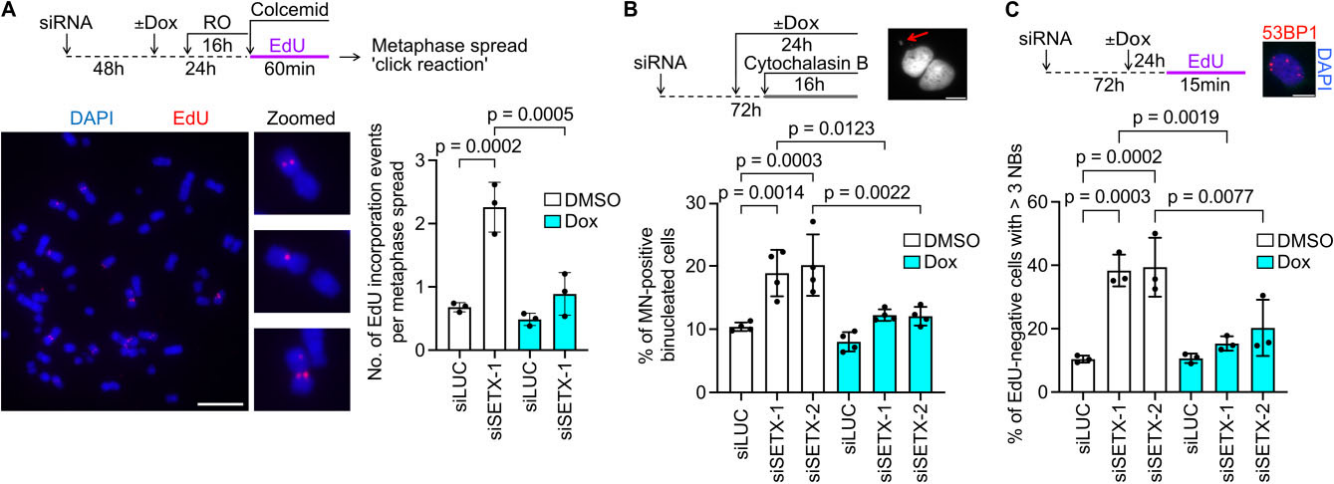
SETX depletion induces R-loop-dependent mitotic DNA synthesis and micronucleation. (**A**) Schematic representation of MiDAS assay, representative images and quantification of EdU incorporation events on metaphase chromosome spreads of U2OS T-REx [RNaseH1-GFP] cells transfected with indicated siRNAs. Cells were treated with 9 $\mu$M CDK1 inhibitor RO-3306 for 16 h, and then released into mitosis in the presence of 20 $\mu$M EdU and colcemid. Where indicated, over-expression of RNaseH1 was induced by the addition of doxycycline (Dox; 1 ng/ml) for 24 h. Dimethylsulphoxide (DMSO) was used as a vehicle. Data represent mean ± SD (*n* = 3). At least 50 metaphase spreads were analyzed for the presence of EdU foci in each experiment. Scale bar, 10 $\mu {\mathrm{m}}.$ (**B**) Experimental workflow with a representative image and quantification of micronuclei in U2OS T-REx [RNaseH1-GFP] cells transfected with indicated siRNAs. Cytochalasin B (2 $\mu$g/ml) was added 16 h prior to fixation to arrest cells before cytokinesis. Dox (1 ng/ml) was added to cells to induce RNaseH1-GFP over-expression. Data represent mean ± SD (*n* = 4). At least 300 binucleated cells were scored in each experiment. Scale bar, 10 $\mu {\mathrm{m}}.$ Red arrow indicates micronucleus. (**C**) Experimental workflow with a representative image and quantification of 53BP1 nuclear bodies in U2OS T-REx [RNaseH1-GFP] cells transfected with indicated siRNAs. EdU incorporation was used to exclude S-phase cells from quantification. Dox (1 ng/ml) was added to cells to induce RNaseH1-GFP over-expression. Data represent mean ± SD (*n* = 3). At least 300 EdU-negative cells were analyzed in each experiment. Scale bar, 10 $\mu {\mathrm{m}}.$ Statistical analysis: Ordinary one-way ANOVA followed by Šídák's multiple comparisons, with a single pooled variance was used in (A)–(C).

Cells experiencing persistent replication stress are known to bypass the S/G2 checkpoints and enter mitosis, where attempted condensation of the under-replicated loci exposes them to nucleolytic cleavage that triggers DNA repair synthesis, termed mitotic DNA synthesis (MiDAS) (36). Recent reports provide evidence for R-loops as a major trigger of MiDAS (10,37). Since SETX prevents R-loop-mediated replication stress, we found it interesting to test the occurrence of MiDAS upon SETX-depletion. Indeed, we observed that SETX depletion led to an increase in the frequency of MiDAS events in U2OS T-REx cells (Figure 2A). Importantly, this phenotype was suppressed upon over-expression of RNaseH1 (Figure 2A), suggesting that R-loop accumulation in SETX-depleted cells leads to under-replicated DNA which then induces MiDAS in early mitosis.

Under-replicated DNA presents a major challenge for cells as its persistence is associated with chromosome segregation defects. Improper chromosome segregation leads to micronucleation, where part of a chromosome is ‘broken’ and gets evicted from the nucleus (38). These micronuclei are considered as hallmarks of genome instability, therefore, we found it necessary to evaluate their occurrence upon SETX depletion. We observed a significant increase in micronucleus frequency in SETX-depleted cells, which was abrogated upon over-expression of RNaseH1, suggesting that it is caused by R-loops (Figure 2B). Daughter cells resulting from replication-defective cells also often contain broken DNA ends in their nuclei, which are readily bound by the 53BP1 protein (39). We found that these structures, called 53BP1 nuclear bodies, were increased in the non-replicating population of cells (EdU-negative) after SETX depletion and this was abrogated upon over-expression of RNaseH1 (Figure 2C).

Together, these data highlight the importance of SETX in promoting replication fork progression in the face of R-loops.

### SETX is essential for restarting R-loop-stalled forks *via* MUS81-LIG4-ELL axis

**Figure 3.**
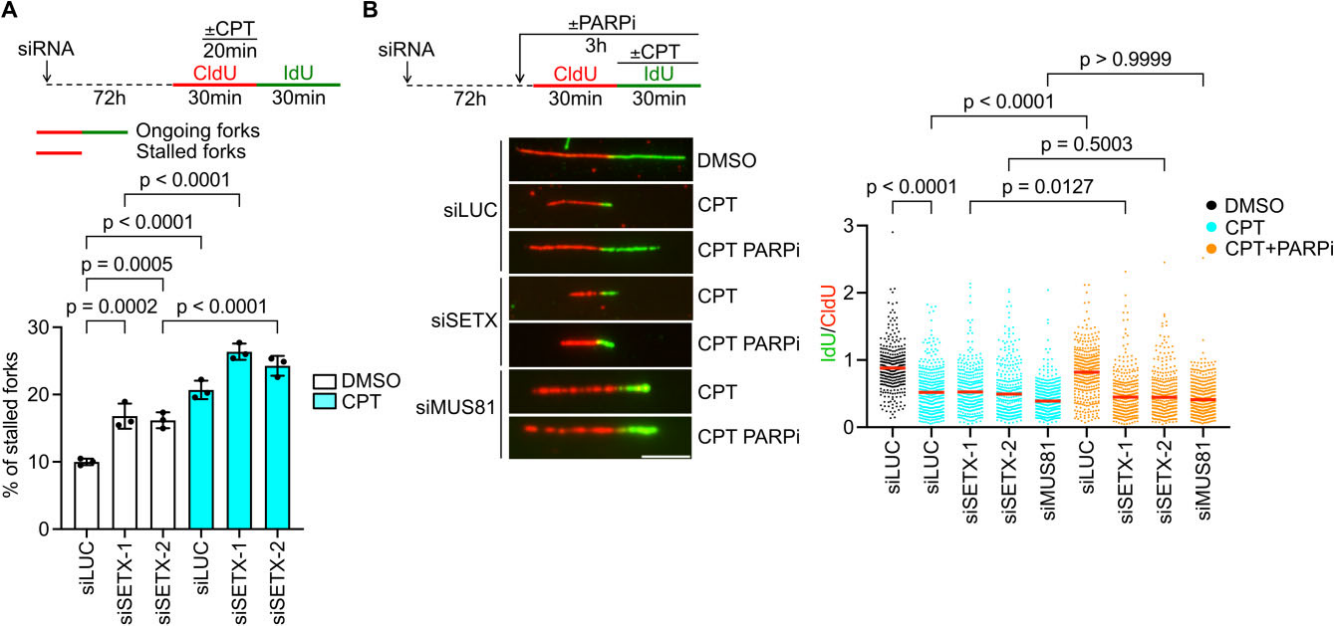
SETX is essential for restarting R-loop-stalled forks *via* MUS81-LIG4-ELL axis. (**A**) Effect of SETX depletion on replication fork recovery after induction of R-loop-mediated fork stalling in U2OS cells. Schematic of DNA fiber assays is shown on the top. The R-loop-inducing drug, camptothecin (CPT; 100 nM) was added to cells during CldU incorporation for the last 20 min. Dimethylsulphoxide (DMSO) was used as a vehicle. Cells were then washed and released into a fresh medium containing IdU. Replication tracts containing only CldU (red-only tracts) were designated as stalled replication forks. Replication tracts containing both CldU and IdU were scored as ongoing forks (red/green tracts). The percentage of stalled replication forks is plotted. Data represent mean ± SD (*n* = 3). (**B**) Effect of SETX depletion on the rescue of CPT-induced fork stalling by PARP inhibition in U2OS cells, which requires the MUS81-LIG4-ELL axis. Schematic of DNA fiber assays and representative images of DNA replication tracts upon indicated conditions are shown on the left. CPT (100 nM) was present during IdU labeling. The PARP inhibitor (PARPi), olaparib (10 $\mu$M) was added 2 h before DNA fiber labeling and was also present during the labeling to boost replication restart. The values of the IdU/CldU tract length ratio obtained in three independent experiments are plotted (*n* > 300). Red lines represent the median. Statistical analysis: Ordinary one-way ANOVA followed by Šídák's multiple comparisons, with a single pooled variance was used in (A). Kruskal–Wallis test followed by Dunn's multiple comparisons test was used in (B). Scale bar, 10 $\mu$m.

The S-phase and mitotic phenotypes of SETX-depleted cells prompted us to examine whether SETX is involved in restarting of R-loop-stalled replication forks. For this, we employed DNA fiber assay where R-loop-mediated replication fork stalling was induced by the DNA topoisomerase I inhibitor, camptothecin (CPT) (10). CPT was added to CldU-incorporating cells, which were then released into drug-free medium containing IdU (Figure 3A, top panel). We observed that SETX-depleted cells had impaired recovery of CPT-stalled replication forks as evident from the measurement of IdU tract length (Supplementary Figure S2A) as well as the frequency of fork stalling events during CldU incorporation (Figure 3A). Our recent work has demonstrated that R-loop-mediated replication fork stalling is elevated in cells exposed to the ribonucleotide reductase inhibitor hydroxyurea (HU) (9). Therefore, we also analyzed the effect of SETX depletion on replication recovery after exposure of cells to a high dose of HU, which, in addition to R-loop-mediated fork stalling, causes replication arrest due to dNTP depletion (9). We found that SETX-depleted cells exhibit a higher frequency of fork stalling events after HU treatment compared to mock-depleted cells (Supplementary Figure S2B). Importantly, this phenotype was fully suppressed upon over-expression of RNaseH1, suggesting again a role for SETX in restarting R-loop-stalled forks (Supplementary Figure S2B).

R-loop-mediated fork stalling leads to replication fork reversal that is counteracted by RECQ1-mediated reverse branch migration triggering replication restart *via* the MUS81-LIG4-ELL axis (10). This can be boosted artificially by inhibiting PARP1, which activates the RECQ1 helicase by eliminating the inhibitory effect of RECQ1 PARylation (11,40). We used this knowledge to further explore the involvement of SETX in the restart of R-loop-stalled forks. In these experiments, CPT was added to cells during the second DNA fiber labeling with IdU to induce R-loop-mediated fork stalling after initiation of DNA replication, allowing the effect of PARP inhibition to be evaluated (Figure 3B, top panel). As expected, we observed that PARP inhibition with olaparib prevented CPT-induced fork stalling in control cells (Figure 3B). However, this unrestrained DNA synthesis was attenuated in SETX-depleted cells, as well as in cells lacking MUS81 (Figure 3B). These results suggest a role for SETX in restarting R-loop-stalled forks *via* the MUS81–LIG4–ELL axis.

### SETX depletion induces MUS81-dependent nascent DNA degradation at R-loop-stalled forks

**Figure 4.**
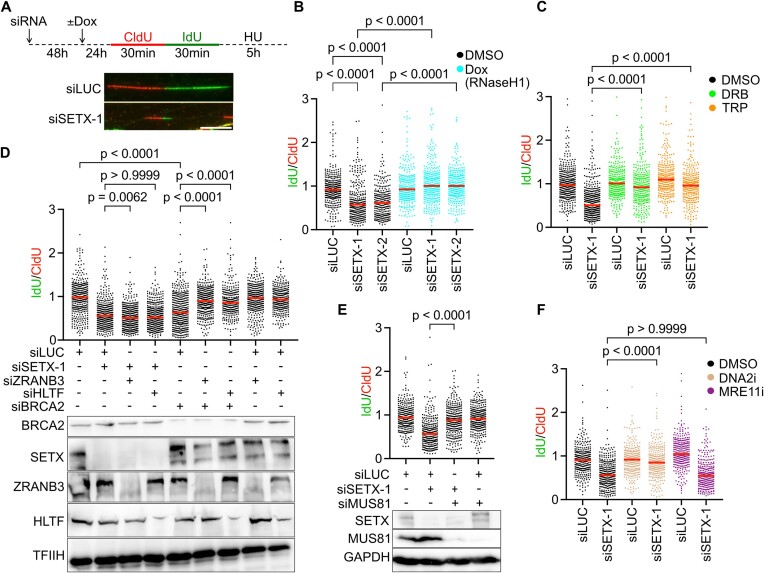
SETX depletion induces MUS81-dependent nascent DNA degradation at R-loop-stalled forks. (**A**) Schematic of fork degradation assay. U2OS T-REx [RNaseH1-GFP] or U2OS cells were transfected with appropriate siRNA and after 72 h were subjected to DNA fiber labeling, followed by hydroxyurea (HU; 4 mM) treatment for 5 h to induce replication fork stalling. Representative images of replication tracts on DNA fibers of mock (siLUC)- and SETX-depleted (siSETX-1) U2OS cells are also shown. Scale bar, 10 $\mu$m. (**B**) Quantification of HU-induced nascent DNA degradation in U2OS T-REx [RNaseH1-GFP] cells transfected with indicated siRNAs. Where indicated, doxycycline (Dox; 1 ng/ml) was added 24 h prior to labeling to induce RNaseH1-GFP over-expression, which eliminates R-loops. (C–F) Quantification of HU-induced nascent DNA degradation in U2OS cells transfected with indicated siRNAs. Where indicated in (**C**), transcription inhibitors, DRB (100 $\mu$M) or triptolide (TRP, 1 $\mu$M), were added 2 h prior to labeling and were present throughout the labeling and HU treatment. In (**D**), SETX or BRCA2 were co-depleted with either ZRANB3 or HLTF to prevent fork reversal. Bottom panel shows western blot to confirm the depletion of indicated proteins. In (**E**), SETX was co-depleted with MUS81 to prevent fork cleavage. Bottom panel shows western blot to confirm the depletion of proteins. Where indicated in (**F**), MRE11 inhibitor, Mirin (50 $\mu$M) and the DNA2 inhibitor, C5 (25 $\mu$M) were added along with HU. In (B)–(F), the values of the IdU/CldU tract length ratio obtained in three independent experiments are plotted (*n* > 300). Red lines represent the median. Statistical analysis: Kruskal–Wallis test followed by Dunn's multiple comparisons test was used in (B)–(F).

In the absence of BRCA2, cells exposed to HU exhibit an extensive MRE11-dependent nascent DNA strand degradation (41), which occurs at R-loop-stalled forks (9). Since we found that SETX is essential for restarting R-loop-stalled forks, we sought to examine the integrity of replication tracts in SETX-depleted cells upon HU treatment. For this, we performed DNA fiber assay where control- and SETX-depleted cells were treated with HU after replication tract labeling with CldU and IdU (Figure 4A). We observed that HU treatment induced extensive shortening of IdU tracts in SETX-depleted cells, which could be rescued by RNaseH1 over-expression or transcription inhibition (Figure 4A–C; Supplementary Figure S3A, B), suggesting R-loop-dependent nascent DNA strand degradation. Nascent DNA strand degradation in BRCA2- or CtIP-depleted cells is dependent on replication fork reversal, where the regressed DNA arm serves as the entry point for nucleases (42,43). Thus, we found it imperative to test if this was also the case for fork degradation in SETX-depleted cells. We inhibited fork reversal by depletion of ZRANB3 or HLTF. Interestingly, depletion of these translocases did not affect fork degradation in SETX-depleted cells, whereas it suppressed fork degradation in BRCA2-depleted cells (Figure 4D). Fork degradation in SETX-depleted cells was also not affected by depletion of the FBH1 helicase (Supplementary Figure S3C), which was implicated in promoting fork degradation in 53BP1-depleted cells by inducing fork remodeling (44). These data indicate that the replication fork degradation in SETX-depleted cells is independent of replication fork reversal. Given the role of SETX in restarting R-loop-stalled forks *via* the MUS81–LIG4–ELL axis, we therefore hypothesized that SETX-deficient cells could accumulate MUS81-cleaved replication forks that undergo nucleolytic resection. Indeed, we found that HU-induced fork degradation in these cells was attenuated by co-depletion of MUS81 (Figure 4E). To identify the nuclease responsible for this nascent DNA strand resection phenotype, fork degradation assays were carried out in the presence of inhibitors of MRE11 (mirin) and DNA2 (C5) nucleases that have been implicated in fork degradation in BRCA2- and CtIP-deficient cells, respectively (41–43). We found that fork degradation in SETX-depleted cells was abrogated by inhibition of DNA2, but not by inhibition of MRE11 (Figure 4F). Together, these results suggest that R-loop accumulation triggers fork degradation in SETX-depleted cells, which is independent of fork reversal, is mediated by DNA2 and initiates from DNA breaks generated by MUS81-mediated fork cleavage. In support of this notion, an extensive fork reversal-independent nascent DNA degradation was also observed in cells lacking LIG4 (Supplementary Figure S4A, B). Importantly, depletion of SETX in LIG4 mutant fibroblasts did not exacerbate fork degradation phenotype (Supplementary Figure S4C), reaffirming that SETX is indeed essential for the restart of R-loop-stalled forks *via* the MUS81–ELL–LIG4 axis.

We also tested whether DNA2-mediated fork degradation plays a role in the observed R-loop-dependent replication fork slowing phenotype of SETX-depleted cells (Figure 1C). For this, U2OS cells were pretreated with the DNA2 inhibitor for 1 hour prior to DNA fiber labeling with CldU and IdU (Supplementary Figure S3D). We found that DNA2 inhibition partially rescued the replication fork slowing phenotype of SETX-depleted cells (Supplementary Figure S3D), suggesting that it results from two consecutive events: (i) fork stalling at an R-loop and (ii) nascent DNA degradation by DNA2 induced by MUS81-mediated fork cleavage.

### Reactivation of R-loop-stalled forks *via* MUS81-LIG4-ELL axis depends on helicase activity of SETX

**Figure 5.**
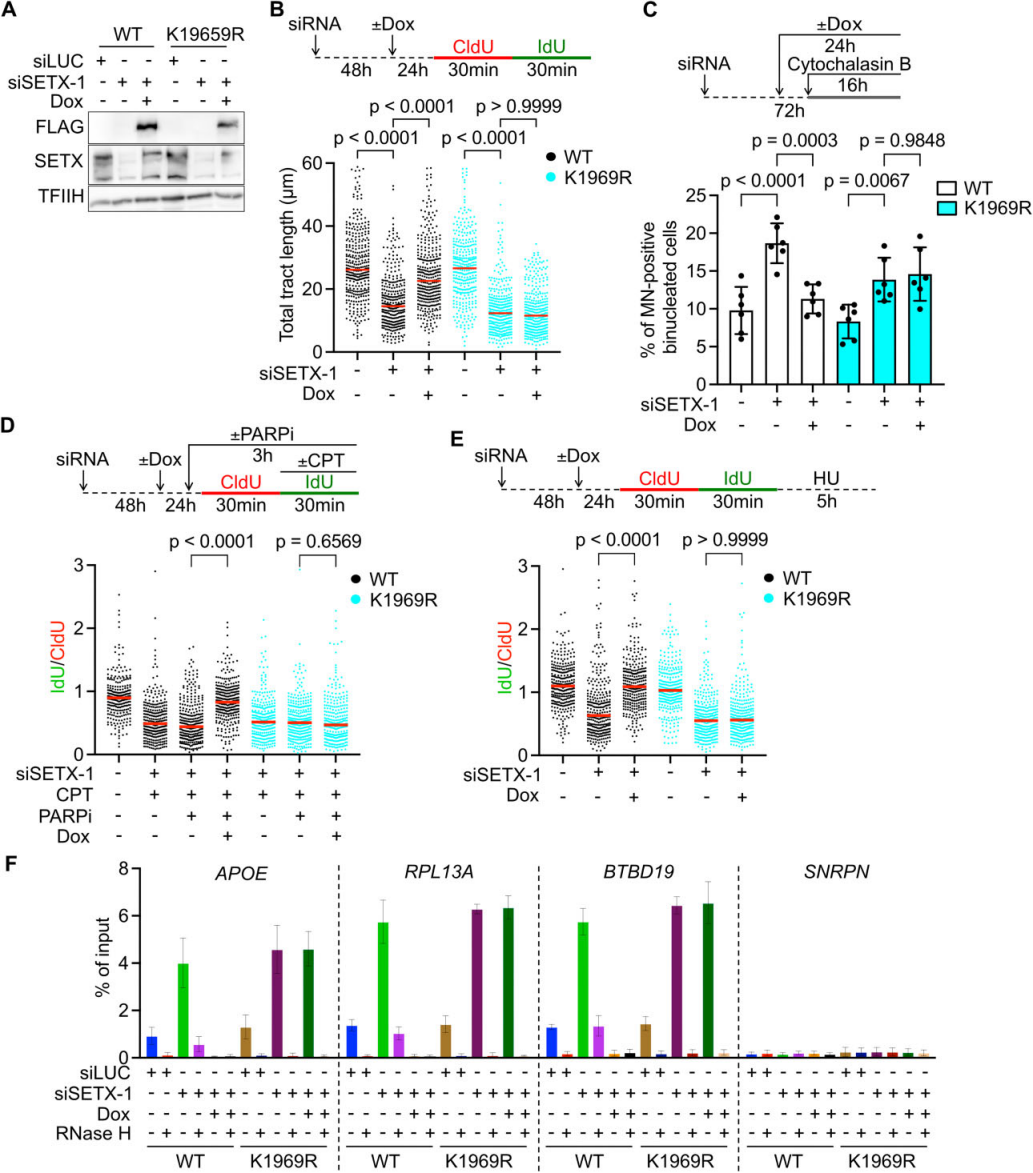
Reactivation of R-loop-stalled forks *via* MUS81–LIG4–ELL axis depends on helicase activity of SETX. (**A**) Western blot analysis of extracts of U2OS T-REx cells carrying siRNA-resistant wild-type (WT) or mutant (K1969R) *SETX* transgenes under the control of doxycycline-regulated CMV promoter. Cells were transfected with siLUC or siSETX-1 and cultured for 72 h. Where indicated, doxycycline (Dox, 10 ng/ml) was added 24 h before harvest to induce transgene expression. TFIIH served as a loading control. (**B**) Effect of expression of WT- or helicase-dead *SETX* transgene on replication fork progression in U2OS T-REx cells depleted of endogenous SETX. The values of CldU + IdU tract lengths measured in three independent experiments are plotted (*n* > 300). Red lines represent the median. (**C**) Micronucleus frequency in U2OS T-REx cells expressing WT- or helicase-dead *SETX* transgene. Cells were transfected with siSETX-1 as in (A) to deplete endogenous SETX. Cytochalasin B was added 16 h prior to fixation to arrest cells before cytokinesis. Data represent mean ± SD (*n* = 6). At least 300 binucleated cells were scored in each experiment. (**D**) Effect of PARP inhibition (PARPi) on CPT-induced fork stalling in U2OS T-REx cells expressing WT- or helicase-dead *SETX* transgene. The values of the IdU/CldU tract length ratio obtained in three independent experiments are plotted (n > 300). Red lines represent the median. (**E**) Effect of expression of WT- or helicase-dead *SETX* transgenes on HU-induced nascent DNA degradation in U2OS T-REx cells depleted of endogenous SETX. The values of the IdU/CldU tract length ratio obtained in three independent experiments are plotted (n > 300). Red lines represent the median. (**F**) R-loop levels in U2OS T-REx cells expressing the WT- or helicase-dead *SETX* transgene. S9.6 antibody was used to pull-down DNA fragments containing RNA:DNA hybrids and the enrichment of R-loop-prone loci (APOE, RPL13A and BTBD19) was measured by qPCR. SNRPN locus was used as a negative control for R-loop enrichment. Data represent mean ± SD (*n* = 4). Statistical analysis: Kruskal–Wallis test followed by Dunn's multiple comparisons test was used in (B), (D) and (E). Ordinary one-way ANOVA followed by Šídák's multiple comparisons, with a single pooled variance was used in (C).

SETX contains a conserved helicase domain, which is required for SETX-mediated unwinding of RNA:DNA hybrids *in vitro* (17,45). To determine whether SETX exerts its function in counteracting R-loop-mediated replication stress through its helicase activity, we performed a phenotypic analysis of cells expressing a SETX variant carrying an arginine substitution at the conserved lysine residue (K1969) in helicase motif I, which is essential for the ATP-dependent helicase activity of the enzyme (45). For this, we used site-directed mutagenesis to produce an siRNA-resistant version of the *SETX* gene in the pAIO vector (30), with or without the desired K1969R mutation. These constructs stably transfected in U2OS T-REx cells allowed us to ectopically express wild-type or mutated SETX from a doxycycline-regulated CMV promoter and to downregulate expression of the endogenous SETX by transfecting the cells with specific siRNA (Figure 5A). As expected, depletion of endogenous SETX reduced replication fork velocity and induced micronucleation in both U2OS T-REx cell lines grown under unchallenged conditions in absence of doxycycline (Figure 5B, C). These cells also exhibited nascent DNA degradation upon HU treatment and a failure to restart CPT-stalled forks upon PARP inhibition (Figure 5D, E). Importantly, all these phenotypes were rescued by ectopic expression of wild-type SETX but not of its helicase mutant (Figure 5B–E), suggesting the importance of SETX helicase activity in promoting the reactivation of R-loop-stalled forks *via* the MUS81–LIG4–ELL axis. To examine whether the helicase activity of SETX plays a role in the suppression of R-loop accumulation, the above cell lines were subjected to DNA–RNA immunoprecipitation (DRIP) experiments using S9.6 antibody, which specifically recognizes RNA:DNA hybrids. The immunoprecipitated material was tested by qPCR for the enrichment of DNA fragments containing specific R-loop-prone regions identified in the APOE, RPL13A and BTBD19 genes (46). The SNRPN gene was used as a negative control. As expected, we observed that SETX depletion increased RNA:DNA hybrid levels at all R-loop-prone loci tested in both cell lines (Figure 5F). Ectopic expression of wild-type SETX abrogated this phenotype, whereas ectopic expression of the helicase-dead mutant did not (Figure 5F). These data accentuate the importance of the helicase activity of SETX in suppressing R-loop-mediated replication stress.

### SETX acts in a common pathway with DDX17 to suppress R-loop-mediated replication stress

**Figure 6.**
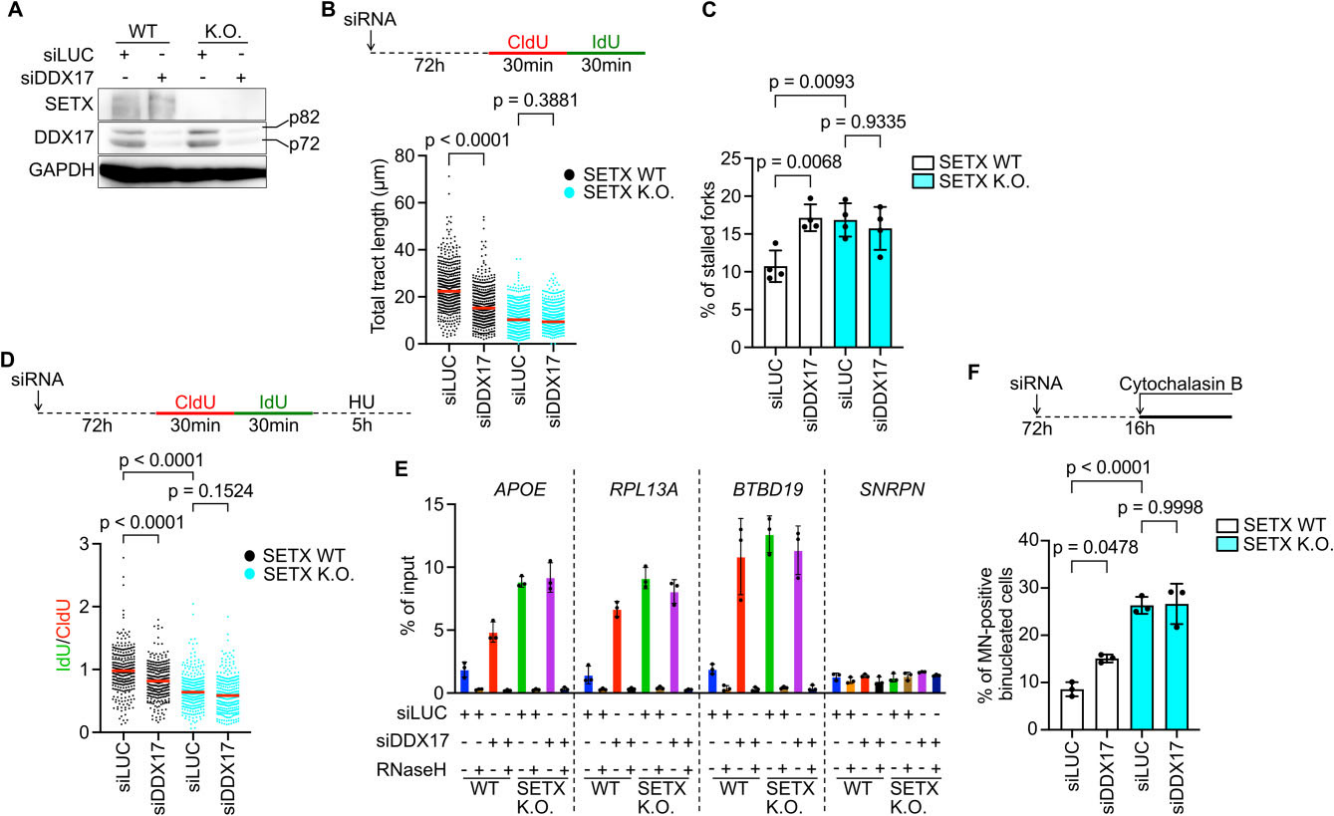
Epistatic relationship between SETX and DDX17 in resolution of R-loop-mediated replication stress. (**A**) Western blot analysis to confirm depletion of both isoforms of DDX17, p72 and p82, in wild-type (WT) and *SETX* knockout U2OS cells, 72 h post siRNA transfection. (**B**) Effect of DDX17 depletion on replication fork progression in WT and *SETX* knockout U2OS cells. Schematic of DNA fiber assay is shown on the top. The values of CldU + IdU tract lengths measured in three independent experiments are plotted (*n* > 300). Red lines represent the median. (**C**) The frequency of stalled replication forks in cells in (B). CldU replication tracts that are not followed by an IdU tract were considered as stalled forks. Data represent mean ± SD (*n* = 4). (**D**) Effect of DDX17 depletion on nascent DNA degradation in WT and *SETX* knockout U2OS cells following HU (4 mM) treatment. Schematic of the fork degradation assay is shown on the top. The values of the IdU/CldU tract length ratio obtained in three independent experiments are plotted (*n* > 300). Red lines represent the median. (**E**) Effect of DDX17 depletion of R-loop levels in WT and *SETX* knockout U2OS cells. S9.6 antibody was used to pull-down DNA fragments containing RNA:DNA hybrids and the enrichment of R-loop-prone loci (APOE, RPL13A and BTBD19) was measured by qPCR. SNRPN locus was used as a negative control for R-loop enrichment. Data represent mean ± SD (*n* = 3). (**F**) Effect of DDX17 depletion on micronucleus frequency in WT and *SETX* knockout cells. Cytochalasin B was added 16 h prior to fixation to arrest cells before cytokinesis. Data represent mean ± SD (*n* = 3). At least 300 binucleated cells were scored in each experiment. Statistical analysis: Kruskal–Wallis test followed by Dunn's multiple comparisons test was used in (B) and (D). Ordinary one-way ANOVA followed by Šídák's multiple comparisons, with a single pooled variance was used in (C) and (F).

Our data establish SETX as an RNA/DNA helicase that eliminates R-loops at sites of R-loop-mediated TRCs to facilitate replication restart *via* the MUS81–LIG4–ELL axis. However, our recent report suggests a similar function for another RNA/DNA helicase, DDX17 (14). Therefore, we investigated if SETX and DDX17 collaborate to ameliorate R-loop-mediated replication stress. To conduct this epistasis analysis, we established a *SETX* knockout U2OS cell line using CRISPR-Cas9-mediated gene editing, and used siRNA to deplete DDX17 (Figure 6A; Supplementary Figure S5A). As expected, disruption of the *SETX* gene was found to induce replication fork stalling, accumulation of RNA:DNA hybrids at R-loop-prone loci, and MUS81/DNA2-dependent nascent DNA degradation upon HU treatment (Figure 6B–E; Supplementary Figure S5B–D). Importantly, these phenotypes of *SETX* knockout U2OS cells were not further exacerbated by the depletion of DDX17 (Figure 6B–E). In addition, DDX17 depletion did not further increase micronucleation in *SETX* knockout cells, whereas it induced this genome instability phenotype in normal U2OS cells (Figure 6F). Furthermore, we found that the replication fork slowing phenotype of *SETX* knockout cells was rescued by ectopic expression of SETX, confirming that it is caused by SETX deficiency (Supplementary Figure S5E). Taken together, these data suggest that SETX and DDX17 act in a common pathway to protect cells against R-loop-mediated replication stress.

### SETX forms a complex with DDX17 in human cells

**Figure 7.**
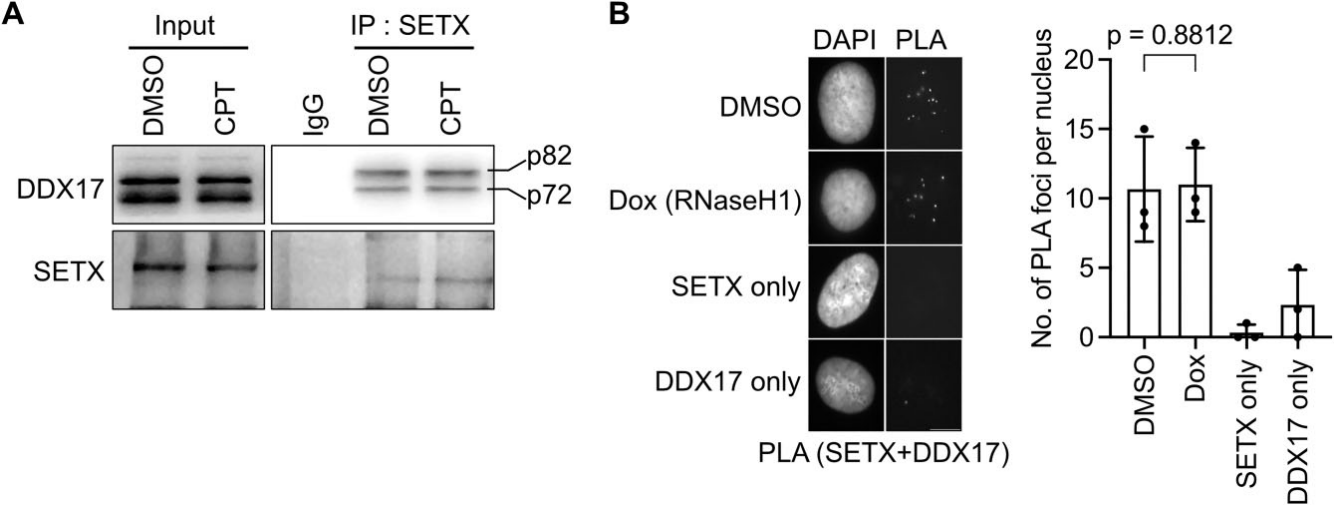
SETX forms a complex with DDX17 in human cells. (**A**) DDX17 isoforms (p72 and p82) co-immunoprecipitate with SETX from HEK293 cell extracts. Cells were treated either with 100 nM CPT or with vehicle (DMSO) for 1 h before harvest. Cell extracts were immunoprecipitated with rabbit polyclonal anti-SETX antibody. Rabbit IgG was incubated with extract of DMSO-treated cells as a control. Representative western blot of three independent experiments is shown. (**B**) Representative images (left panel) and quantification (right panel) of PLA foci between SETX and DDX17 in U2OS T-REx [RNaseH1-GFP] cells before and 24 h after induction of RNaseH1 expression with doxycycline (Dox). Medians of data sets from three independent experiments are plotted (n > 200). Data are presented as mean ± SD. Statistical analysis: Ordinary one-way ANOVA followed by Šídák's multiple comparisons, with a single pooled variance. Scale bar, 10 *μ*m.

To test whether SETX and DDX17 interact physically, we immunoprecipitated SETX from HEK293 cell extracts that had been pretreated with benzonase to eliminate any nucleic acid-mediated protein complexes. Western blot analysis showed that both DDX17 isoforms (p72 and p82) were co-immunoprecipitated with the SETX antibody, but not with the control IgG (Figure 7A). This suggests that SETX and DDX17 form a complex *in vivo*. The treatment of cells with CPT did not results in an increase in the cellular level of this complex, suggesting that its formation is not dependent on the presence of R-loops (Figure 7A). In accordance with this hypothesis, we observed by PLA that SETX and DDX17 colocalized in the nuclei of U2OS T-REx [RNaseH1-GFP] cells even upon RNaseH1 overexpression (Figure 7B).

## Discussion

R-loop-associated head-on TRCs present a major source of genome instability (3,47). Our earlier work has shown that replication forks stalled at R-loops are cleaved by MUS81-EME1 endonuclease, paving the way for replication restart (10). In this study, we provide evidence that SETX aids replication fork restart *via* the MUS81–ELL–LIG4 axis. We report that SETX is essential for R-loop resolution specifically in the S-phase of the cell cycle, suggesting its replication-coupled role. This finding is in line with a similar observation for the yeast SETX homolog, Sen1, which was recently reported to be an R-loop-resolving helicase specific to S-phase (48). Our experiments with the helicase-dead mutant (K1969R) of SETX further emphasized the notion that R-loop resolution by SETX is indispensable for normal DNA replication fork progression.

R-loop-dependent degradation of nascent DNA in response to HU treatment was another notable phenotype of SETX-depleted cells or cells expressing the SETX helicase mutant. Lack of requirement for fork reversal and dependence on MUS81 indicated that nascent DNA strand degradation in SETX-deficient cells initiates from cleaved replication forks. We envisage that lack of SETX, or any other factor acting downstream of MUS81 in the MUS81–ELL–LIG4 replication fork restart pathway will lead to accumulation of MUS81-cleaved replication forks that will undergo nucleolytic resection. Indeed, we found that cells depleted of LIG4, another factor in the above replication restart pathway, accumulate MUS81-dependent DSBs (10), and exhibit a similar fork degradation phenotype as SETX-depleted cells (Supplementary Figure S4B). Moreover, SETX depletion did not further exacerbate the nascent DNA strand degradation phenotype of LIG4 mutant fibroblasts (Supplementary Figure S4C). Thus, the replication fork degradation assay established in this study could be used to evaluate the involvement of other candidate factors in the MUS81–ELL–LIG4 pathway.

**Figure 8.**
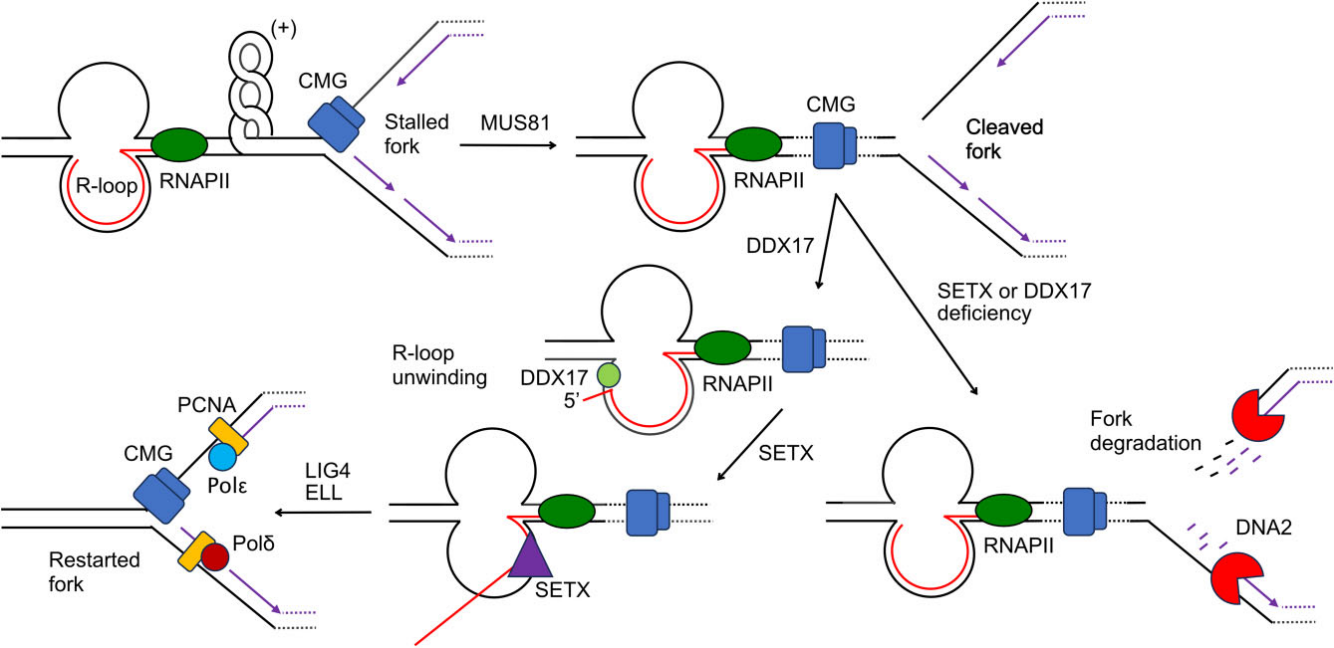
Model for the role of SETX in restarting R-loop-stalled forks *via* the MUS81–LIG4–ELL pathway. To resume DNA replication, the stalled replication fork is cleaved by MUS81 endonuclease, which results in resolution of the torsional stress in the DNA generated by the transcription-replication encounter. SETX is proposed to act in a sequential manner with DDX17 helicase, possibly as a SETX-DDX17 complex (not shown), to mediate R-loop unwinding, a prerequisite for RNAPII transcription restart. In this process, DDX17 partially unwinds the RNA:DNA hybrid generating a 5′-single-standed RNA tail where SETX is loaded to initiate processive unwinding of the RNA:DNA hybrid. DNA replication resumes following fork religation by DNA ligase IV (LIG4) and ELL-mediated reactivation of transcription. SETX or DDX17 deficiency leads to accumulation of MUS81-cleaved forks, which are subjected to DNA2-mediated nucleolytic processing resulting in genomic instability. CMG, CDC45–MCM2-7–GINS helicase; Polϵ, DNA polymerase epsilon; Polδ, DNA polymerase delta; RNAPII, RNA polymerase II.

Interestingly, our recent study identified the DEAD-box helicase DDX17 as another RNA/DNA helicase that can unwind R-loops *in vitro*, and is also required for replication fork restart *via* the MUS81–ELL–LIG4 pathway (14). Epistasis analysis conducted in the current study using *SETX* knockout cells revealed that SETX and DDX17 act in the same pathway to resolve R-loop-mediated replication stress (Figure 6). Moreover, we found that SETX and DDX17 form a complex in human cells (Figure 7). Thus, it is possible that these two RNA/DNA helicases act on R-loops at TRC sites in a concerted fashion to mediate an efficient R-loop resolution, a prerequisite for replication restart *via* the MUS81–LIG4–ELL axis. Of note, the helicase domain of SETX was shown to require a 5′-RNA overhang to efficiently unwind oligonucleotide-based R-loops *in vitro* (17). On the contrary, DDX17 could efficiently unwind oligonucleotide-based R-loops without RNA overhangs (14), most likely through direct loading of the enzyme onto RNA:DNA duplex aided by its binding to the proximal ssDNA loop in the R-loop structure, a mechanism proposed for DEAD-box helicases (15). Thus, DDX17 might act on the R-loops first, generating a 5′-overhang for SETX to initiate ATP-dependent translocation along RNA and complete R-loop unwinding (Figure 8). It will be interesting to test this model using purified proteins and plasmid-based R-loop structures generated by *in vitro* transcription, which are similar in length to R-loop structures observed in cells (16).

SETX is known to be recruited to transcription pause sites by BRCA1, where it alleviates R-loop-dependent DNA damage (24). Whether the BRCA1-SETX interaction plays a role in the DNA replication-associated function(s) of SETX is an interesting avenue of investigation. However, it should be noted that our earlier study has shown that, unlike SETX, BRCA1 is dispensable for restarting DNA replication after exposure of cells to R-loop-inducing drugs such as CPT (10). Moreover, BRCA1 is rather involved in the protection of reversed replication forks, which counteracts replication restart (49).

## Supplementary Material

gkae673_Supplemental_File

## Data Availability

The data underlying this article are available in the article and in its Supplementary material.

## Notes

Present address: Satyajeet Rao, Swiss Institute for Experimental Cancer Research (ISREC), School of Life Sciences, École Polytechnique Fédérale de Lausanne (EPFL), 1015 Lausanne, Switzerland.

Present address: Esin Isik, Department of Oncologic Pathology, Dana-Farber Cancer Institute, 450 Brookline Avenue, Mayer Building 641, Boston, MA 02215, USA.
