# Supplementary material for: Senataxin RNA/DNA helicase promotes replication restart at co-transcriptional R-loops to prevent MUS81-dependent fork degradation
Source: Nucleic Acids Res. 2024 Aug 9;52(17):10355–69. doi: 10.1093/nar/gkae673 (PMC11417401; doi:10.1093/nar/gkae673)
Supplement: gkae673_Supplemental_File [file gkae673_supplemental_file.pdf]

Supplementary Table S1. Primers used for SETX cloning and mutagenesis.

Name	Sequence (5' to 3')	Purpose
SETX Forward	GTAGTATCCGGAATGAGCACATGTTGTT GGTGT	Cloning to pAIO vector
SETX Reverse	GATATCGGTACCTAAAAGCTTTCTTTTCT TGGAACTGC	Cloning to pAIO vector
siR SETX-1 Forward_1	TATGGTGACTTGCAGTCAAATTGTATACA ATTATAAT	Mutagenesis of the first half of siSETX#1 site
siR SETX-1 Reverse_1	ATTATAATTGTATACAATTTGACTGCAAG TCACCATA	Mutagenesis of the first half of siSETX#1 site
siR SETX-1 Forward_2	TTGCAGTCAAATTGTGTATAACTATAATC CTGAAAAG	Mutagenesis of the second half of siSETX#1 site
siR SETX-1 Reverse_2	CTTTTCAGGATTATAGTTATACACAATTT GACTGCAA	Mutagenesis of the second half of siSETX#1 site
SETX K1969R Forward	CACCTGGAACAGGAAGATCAAAAACTAT TGT	Forward primer for SETX K1969R mutagenesis
SETX K1969R Reverse	ACAATAGTTTTTGATCTTCCTGTTCCAGG TG	Reverse primer for SETX K1969R mutagenesis

Supplementary Table S2. Sequences of the sense strand of siRNA duplexes.

Name	Sequence (5’ to 3’)	Source
siLUC	CGUACGCGGAAUACUUCGAdTdT	Microsynth
siSETX-1	GCCAGAUCGUAUACAAUUAdTdT	Microsynth
siSETX-2	GAGAGAAUUAUUGCGUACUdTdT	Microsynth
siMUS81	CAGCCCUGGUGGAUCGAUAdTdT	Microsynth
siBRCA2	CAGGACACAAUUACAACUAAAdTdT	Microsynth
siZRANB3	GAUCAGACAUCACACGAUUdTdT	Microsynth
siHLTF	AACAAGTGAATTGCCGCAGAAdTdT	Microsynth
siDDX17	CCGGGAGCUACCAAUAUGAUAdTdT	Microsynth
siLIG4	GCUAGAUGGUGAACGUAUGdTdT	Microsynth

Supplementary Table S3. Sequences of primers used for DRIP-qPCR.

Name	Sequence (5’ to 3’)	Source
APOE Forward	CCGGTGAGAAGCGCAGTCGG	Microsynth
APOE Reverse	CCCAAGCCCGACCCCGAGTA	Microsynth
RPL13A Forward	AATGTGGCATTTCCTTCTCG	Microsynth
RPL13A Reverse	CCAATTCGGCCAAGACTCTA	Microsynth
BTBD19 Forward	GGCTGCTCAGGAGAGCTAGA	Microsynth
BTBD19 Reverse	ACCAGACTGTGACCCCAAAG	Microsynth
SNRPN Forward	TGCCAGGAAGCCAAATGAGT	Microsynth
SNRPN Reverse	TCCCTCTTGGCAACATCCA	Microsynth

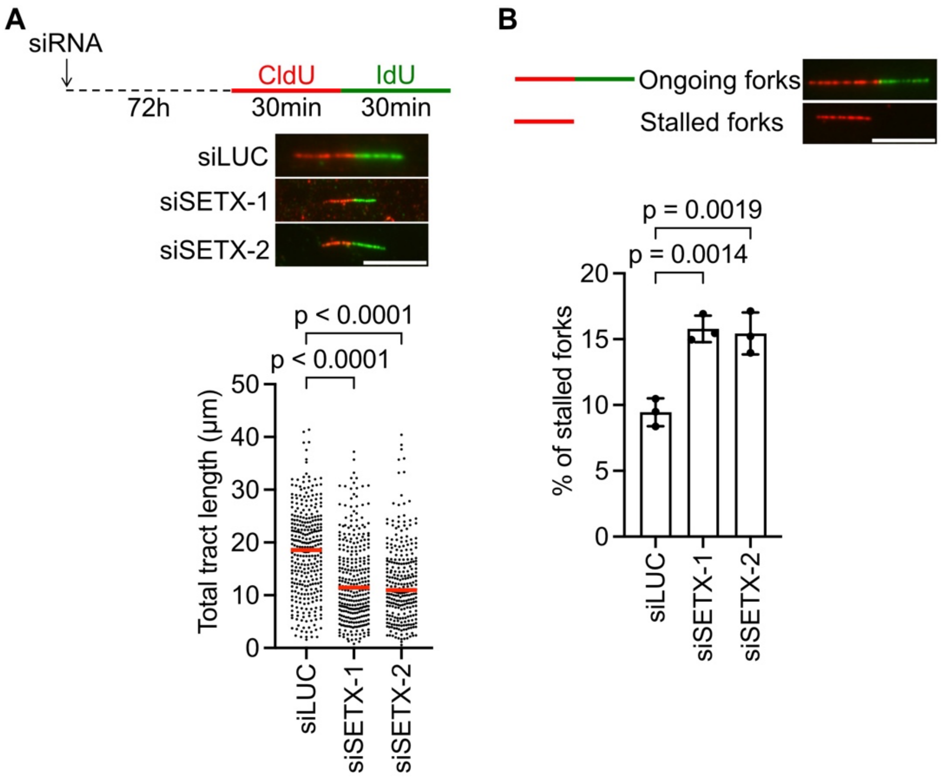

Supplementary Figure S1. SETX depletion induces replication stress. (**A**) Schematic representation of DNA fiber assay, representative images and quantification of replication tracts in mock- (siLUC) and SETX-depleted U2OS cells. The values of CldU+IdU tract lengths of three independent experiments are plotted ($n > 300$). Red lines represent the median. Statistical analysis: Kruskal-Wallis test followed by Dunn's multiple comparisons test. Scale bar, 10 μm. (**B**) Quantification of replication fork stalling events on DNA fibers in (A). Replication tracts containing only the first label (CldU-only tracts) were designated as stalled replication forks, while the replication tracts containing both labels (CldU+IdU) were scored as ongoing forks. Representative images are shown on the top. Percentage of stalled replication forks is plotted for indicated conditions. Data represent mean ± SD ($n = 3$). Statistical analysis: Ordinary one-way ANOVA followed by Šídák's multiple comparisons, with a single pooled variance. Scale bar, 10 μm.

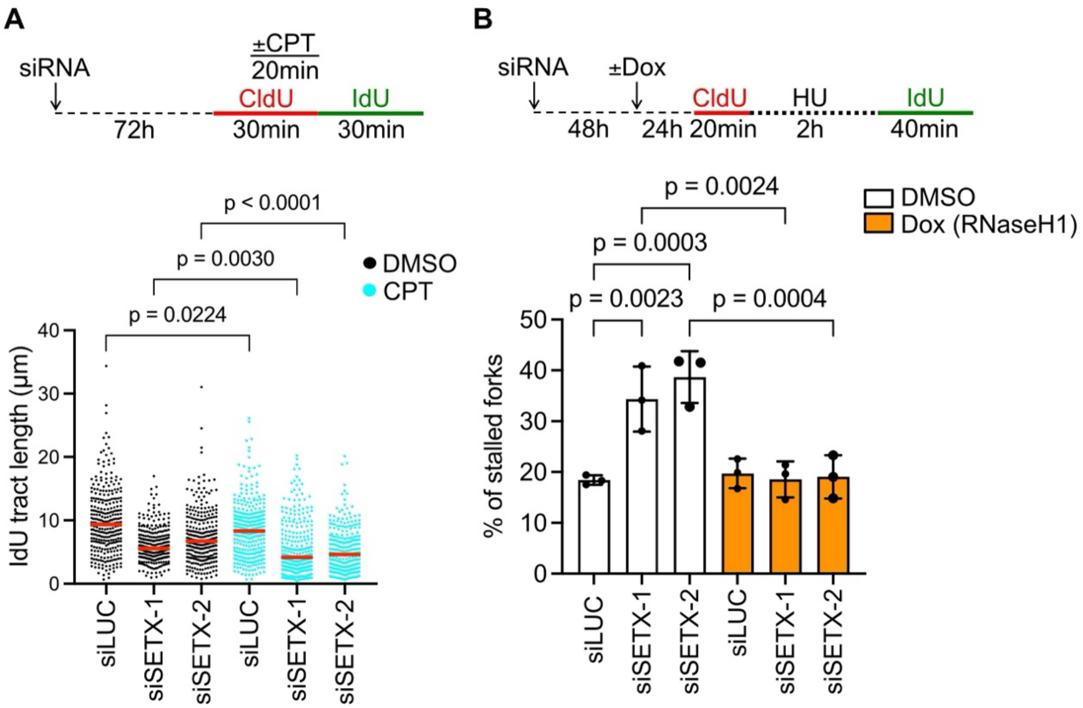

Supplementary Figure S2. SETX is essential for restarting R-loop-stalled forks. (**A**) Effect of SETX depletion on replication fork recovery after exposure of cells to camptothecin (CPT). Schematic of DNA fiber assay is shown on the top. U2OS cells were treated with CPT (100 nM) during CldU labeling for the last 20 min and then washed and released into a fresh medium containing IdU. The values of the IdU/CldU tract length ratio obtained in three independent experiments are plotted (n > 300). Red lines represent the median. Statistical analysis: Kruskal-Wallis test followed by Dunn's multiple comparisons test. (**B**) Effect of SETX depletion on replication fork recovery after exposure of cells to hydroxyurea (HU). Schematic of DNA fiber assay is shown on the top. U2OS T-REx [RNaseH1-GFP] cells were treated with HU (2 mM) for 2 hours after CldU labeling, and then released into fresh medium containing IdU. Where indicated, over-expression of RNaseH1 was induced by addition of doxycycline (Dox; 1 ng/ml) 24 hours before replication tract labeling. Percentage of stalled replication forks (CldU-only tracts) was determined. Data represent mean ± SD (n = 3). Dimethylsulphoxide (DMSO) was used as a vehicle.

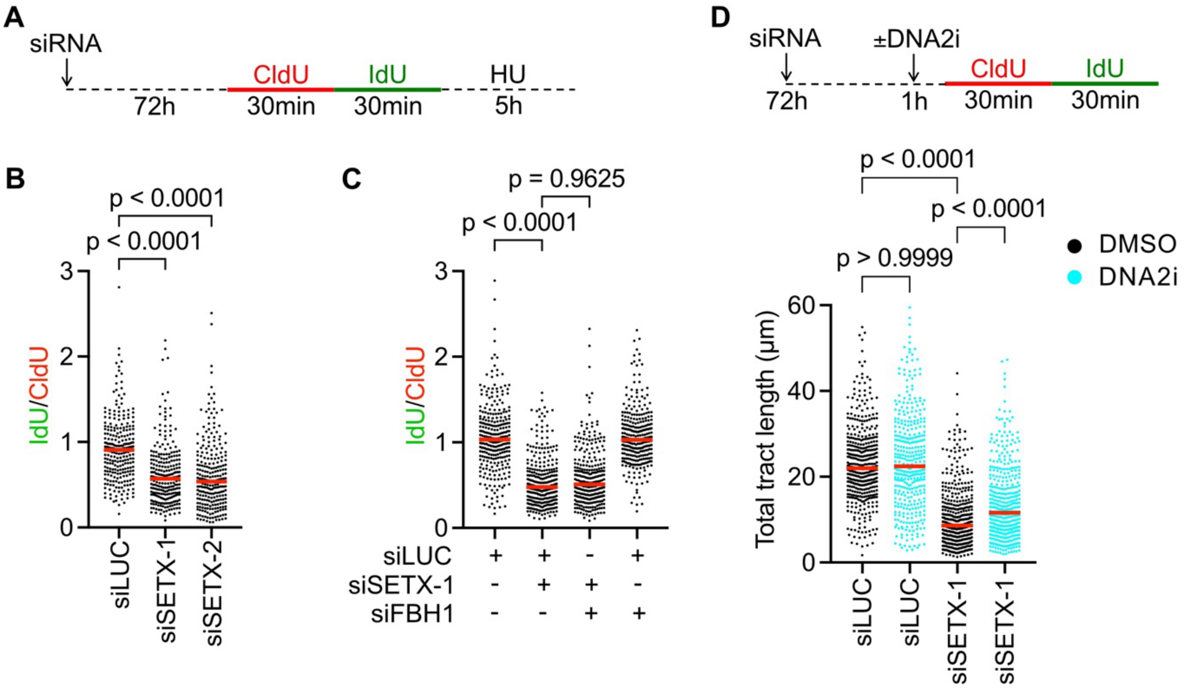

Supplementary Figure S3. SETX depletion induces nascent DNA degradation at R-loop-stalled forks. (**A**) Schematic of fork degradation assay. U2OS cells were transfected with appropriate siRNA and, after 72 hours, were subjected to DNA fiber labeling, followed by hydroxyurea (HU; 4 mM) treatment for 5 hours to induce R-loop-mediated replication fork stalling. (**B, C**) Quantification of HU-induced nascent DNA degradation in cells transfected with indicated siRNAs. The values of the IdU/CldU tract length ratio obtained in two independent experiments are plotted ($n > 250$). The red lines represent the median. (D) Replication fork slowing phenotype of SETX-depleted U2OS cells is caused, in part, by DNA2-dependent fork degradation. Schematic of DNA fiber assay is shown on the top. DNA2 inhibitor (DNA2i), C5 (25 μM) was added 1 hour before DNA fiber labeling and was also present during the labeling. The values of CldU+IdU tract lengths measured in three independent experiments are plotted ($n > 300$). Red lines represent the median. Statistical analysis: Kruskal-Wallis test followed by Dunn's multiple comparisons test was used in (B) to (D).

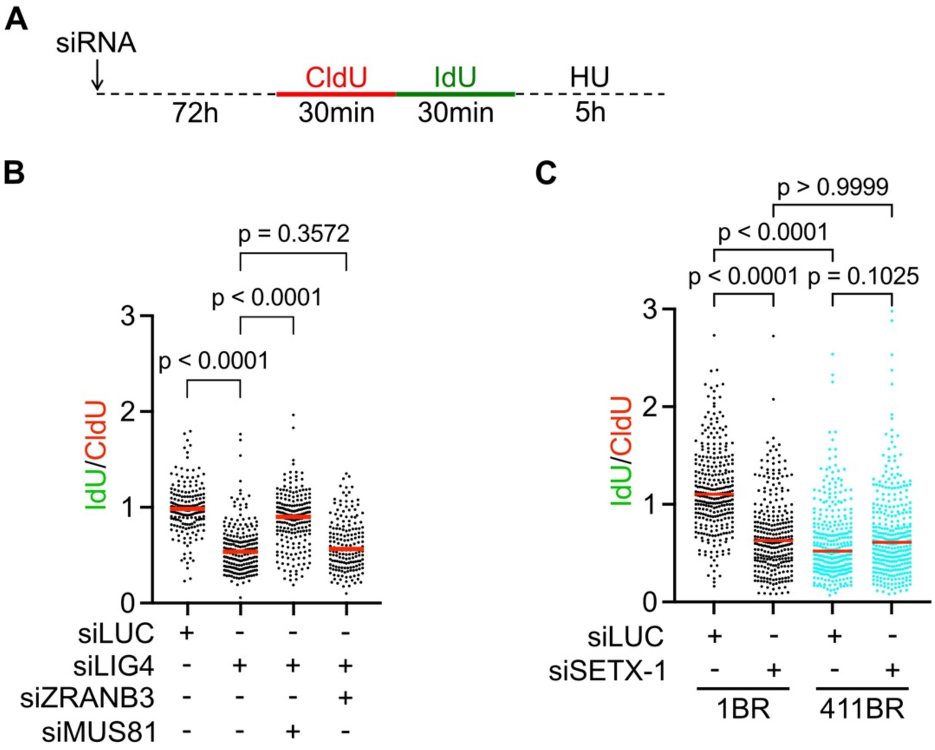

Supplementary Figure S4. LIG4 deficiency induces MUS81-dependent nascent DNA degradation at R-loop-stalled forks. (**A**) Schematic of fork degradation assay. Cells were transfected with appropriate siRNA and, after 72 hours, were subjected to DNA fiber labeling, followed by hydroxyurea (HU; 4 mM) treatment for 5 hours to induce R-loop-mediated replication fork stalling. (**B**) Quantification of HU-induced nascent DNA degradation in U2OS cells transfected with indicated siRNAs. (**C**) Quantification of HU-induced nascent DNA degradation in control (1BR) and patient fibroblasts (411BR) containing a mutation in the catalytic domain of LIG4. Depletion of SETX was achieved by transfection of specific siRNA. (**B, C**) The values of the IdU/CldU tract length ratio obtained in three independent experiments are plotted ($n > 300$). Red lines represent the median. Statistical analysis: Kruskal-Wallis test followed by Dunn's multiple comparisons test.

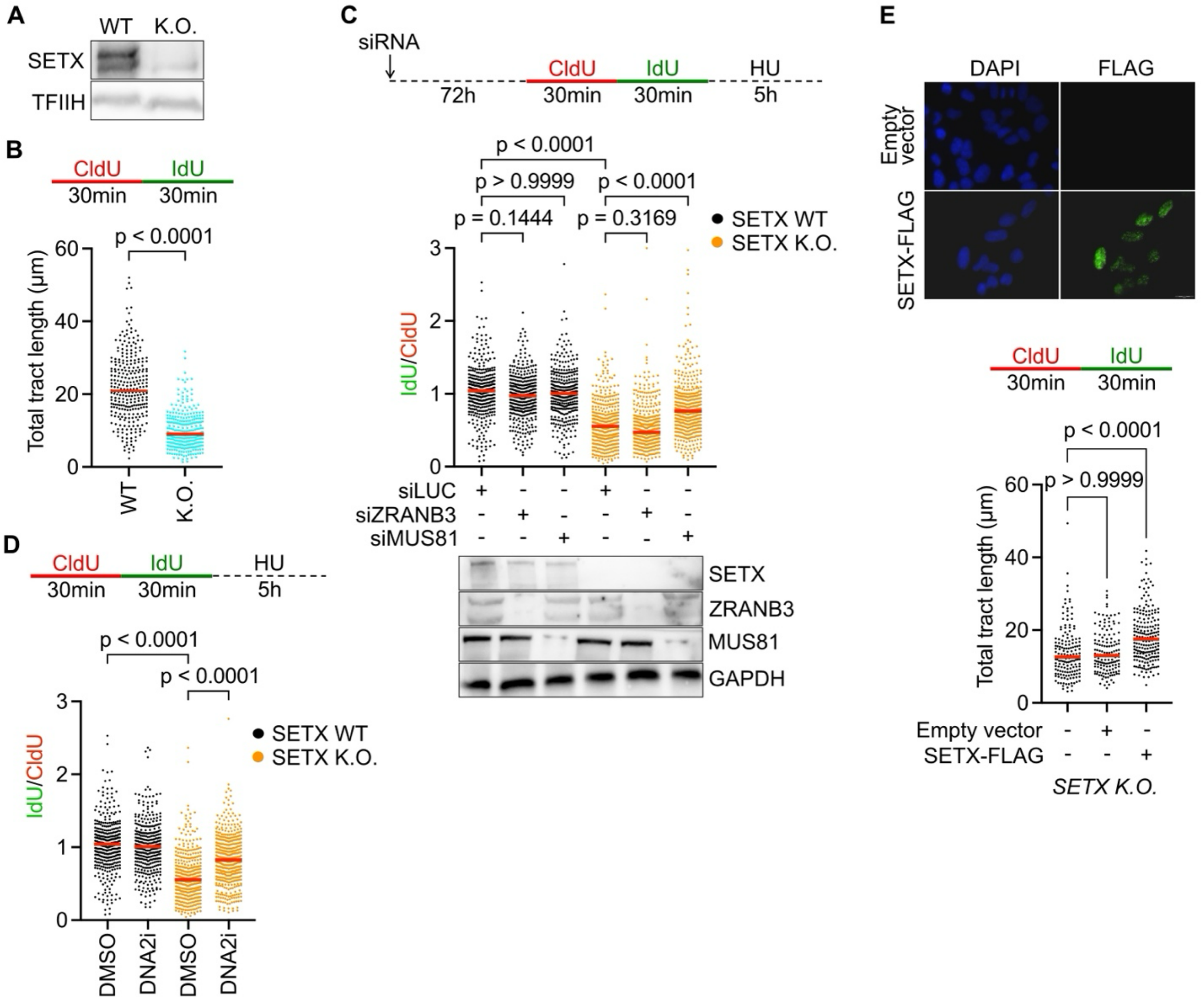

Supplementary Figure S5. Disruption of *SETX* gene induces replication stress in U2OS cells. (**A**) Western blot analysis to confirm *SETX* knockout (K.O.). Exon 3 of the *SETX* gene was targeted for Cas9-mediated cleavage in U2OS cells. (**B**) Disruption of the *SETX* gene slows down the progression of replication forks in U2OS cells. The values of CldU+IdU tract lengths of three independent experiments are plotted for indicated conditions (n > 300). Red lines represent the median. (**C**) Disruption of the *SETX* gene causes MUS81-dependent nascent DNA degradation upon hydroxyurea (HU; 4 mM) treatment. Schematic of fork degradation assay is shown on the top. Depletion of ZRANB3 or MUS81 was achieved by transfection of specific siRNAs. The values of the IdU/CldU tract length ratio obtained in three independent experiments are plotted (n > 300). Red lines represent the median. Bottom panel shows western blot to confirm depletion of proteins. (**D**) HU-induced nascent DNA degradation in *SETX* knockout cells depends on DNA2.

Schematic of fork degradation assay is shown on the top. DNA2 inhibitor, C5 (25 μM) was added along with HU for 5 hours. The values of the IdU/CldU tract length ratio obtained in three independent experiments are plotted (n > 300). Red lines represent the median. **(E)** Complementation of *SETX* knockout cells with *SETX* cDNA. *SETX* knockout cells were transfected with either the pAIO-SETX-FLAG construct or empty vector (pAIO) and single clones were isolated. Top panel: Representative immunofluorescence images of *SETX* knockout cells ectopically expressing *SETX* transgene as detected using anti-FLAG antibody. Scale bar, 20 μm. Bottom panel: Schematic of DNA fiber labeling and quantification of replication tract lengths in parental *SETX* knockout cells and their derivatives harboring *SETX* transgene or empty vector. The values of CldU+IdU tract lengths are plotted for indicated conditions (n > 150). Red lines represent the median. Statistical analysis: Kruskal-Wallis test followed by Dunn's multiple comparisons test in (B) to (E).
